# Supplementary material for: Neoantigen-specific T cell help outperforms non-specific help in multi-antigen DNA vaccination against cancer
Source: Mol Ther Oncol. 2024 Jun 15;32(3):200835. doi: 10.1016/j.omton.2024.200835 (PMC11261851; doi:10.1016/j.omton.2024.200835)
Supplement: Document S1. Figures S1–S4 and Tables S1–S3 [file mmc1.pdf]

OMTON, Volume *32*

Supplemental information

Neoantigen-specific T cell help outperforms non-specific help in multi-antigen DNA vaccination against cancer

Joanna Fréderique de Graaf, Tamara Pesic, Felicia S. Spitzer, Koen Oosterhuis, Marcel G.M. Camps, Iris Zoutendijk, Bram Teunisse, Wahwah Zhu, Tsolere Arakelian, Gerben C. Zondag, Ramon Arens, Jeroen van Bergen, and Ferry Ossendorp

Supplemental information

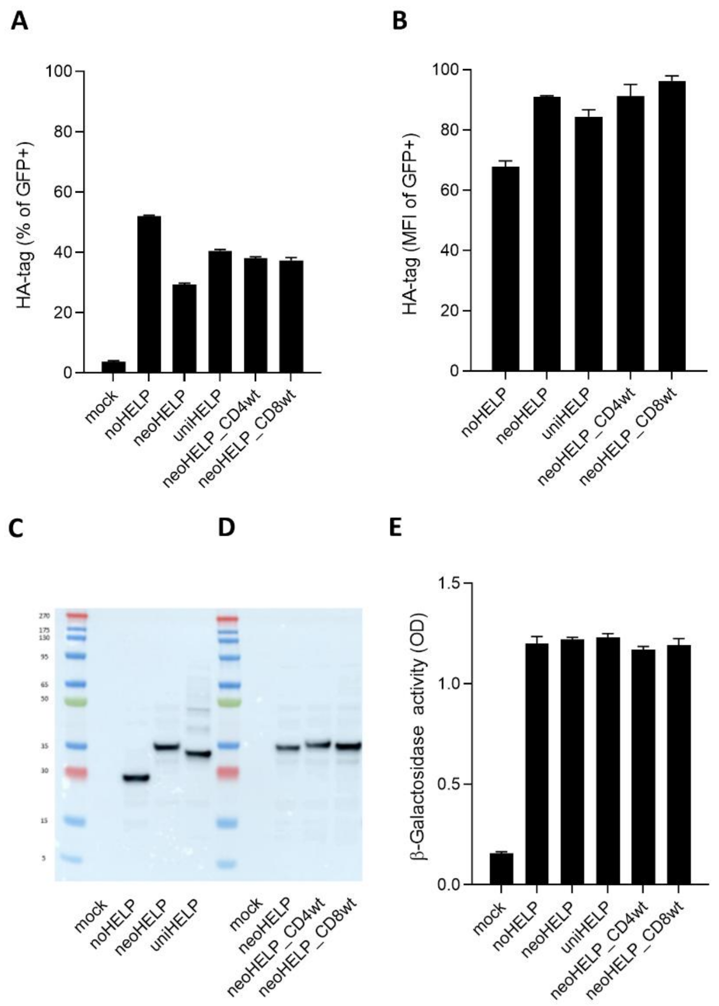

Figure S1. Verification of vaccine constructs. (A-B) HEK293T cells were transfected with a GFP-encoding plasmid in combination with the indicated vaccine-encoding plasmids. Transfected cells were identified by GFP-expression, and a C-terminal HA-tag allowed detection of multi-antigen vaccine proteins. (A) The fraction (%) of HA-positive cells among transfected (GFP^+) cells, and (B) their mean fluorescence intensity (MFI) were determined by flow cytometry. The MFI of mock-transfected cells is not shown as these cells – not having been transfected with a plasmid encoding an HA-tagged protein - do not have detectable expression of the HA-tag above background. Bars and whiskers represent means and standard errors (SEM) of triplicates, respectively. (C-D) Western blot detection of HA-tagged (C) noHELP, neoHELP, uniHELP or (D) neoHELP, neoHELP_CD4wt or neoHELP_CD8wt in HEK293T cells transfected with the indicated vaccine-encoding plasmids. (E) Recognition of OVA antigen on transfected B16-F10 cells by H-$2K^b$/OVA-specific $CD8^+$ T-cell hybridoma B3Z. β-galactosidase expression in B3Z cells is controlled by NFAT, allowing detection of TCR-mediated activation by color conversion of CPRG substrate, which results in increased optical density (OD) at 594nm. Bars and whiskers represent means and standard errors (SEM) of triplicates, respectively. All experiments were performed twice, and one representative experiment is shown.

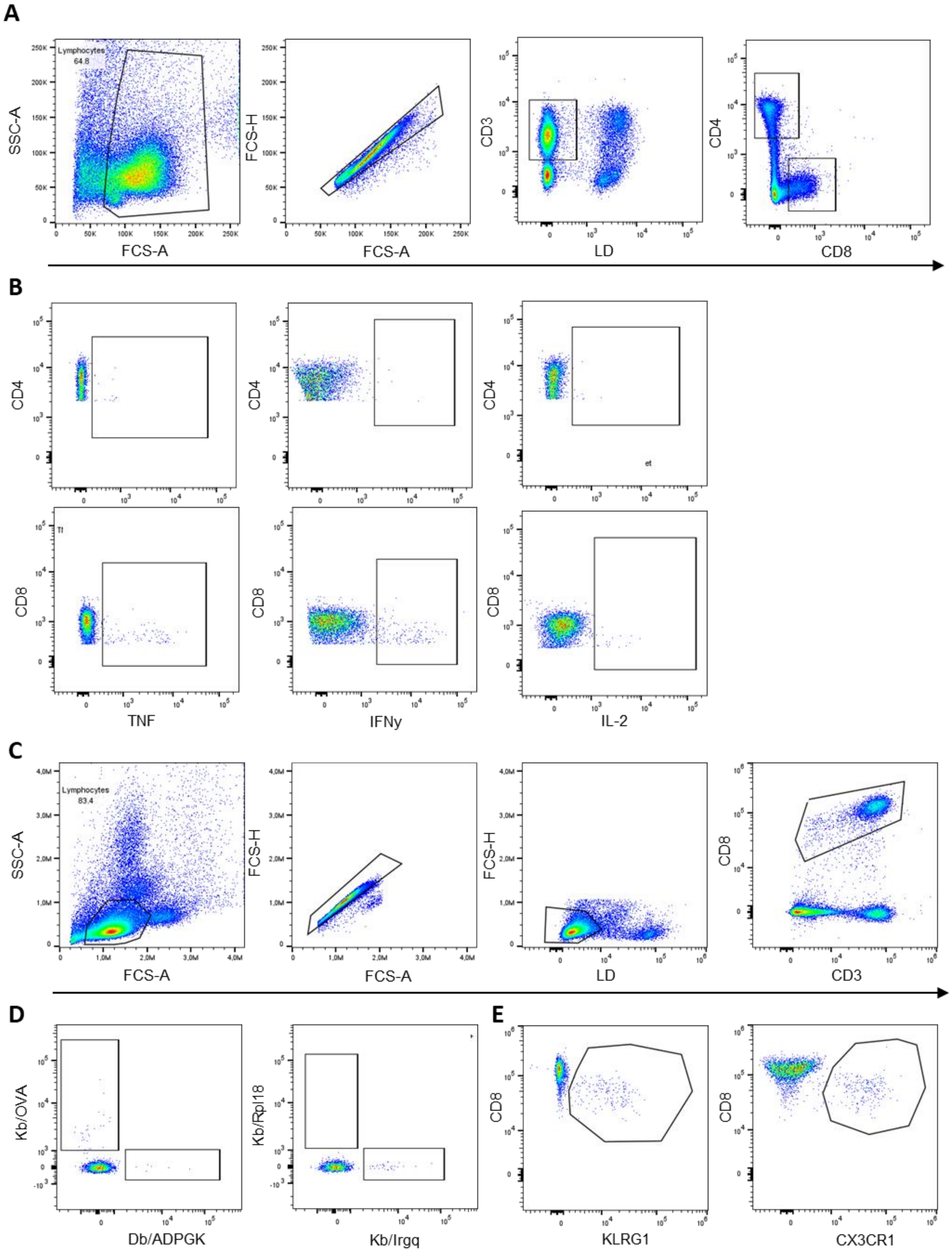

Figure S2. Gating strategy for flow cytometry analysis. (A-B) Gating strategy for spleen-derived T-lymphocytes for ICS. (A) Live CD4⁺ and CD8⁺ T-cells were gated after which (B) TNF-, IFN-γ- and IL-2-positive cells were selected. (C-E) Gating strategy for antigen-specific peripheral blood- and spleen-derived T-lymphocytes for surface marker analysis. (C) Gating of live CD8⁺ T-cells was followed by (D) selection of tetramer-positive cells. (E) Subsequently, cells positive for specific surface markers were identified.

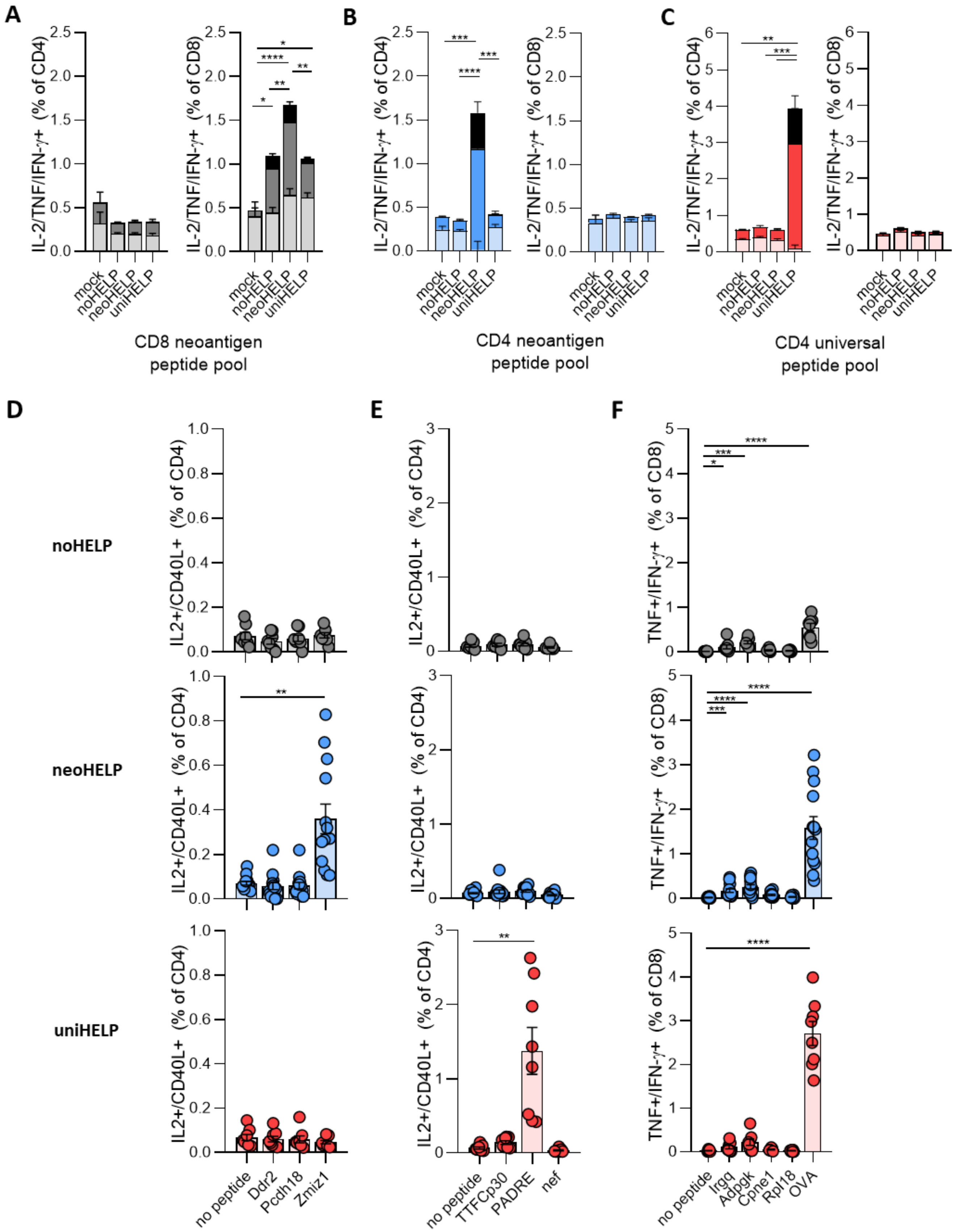

Figure S3. Antigen-specific CD4+ and CD8+ T-cell responses in the spleen of mice. Mice were vaccinated with the indicated vaccines three times, at three-week intervals. Ten days after the final vaccination, spleen cells were cultured with dendritic cells loaded with indicated (A-C) peptide pools or (D-F) individual peptides for 5 hours, and analyzed by intracellular cytokine staining (ICS). IL-2-, TNF-

and/or IFN-γ-positive $CD4^+$ or $CD8^+$ T-cells upon stimulation with the indicated peptide pools: (A) CD8 neoantigen peptide pool (Irgq, Adpgk, Cpne1, Rpl18), (B) CD4 neoantigen peptide pool (Ddr2, Pcdh18, Zmiz1), (C) CD4 universal peptide pool (TTFCp30, PADRE, nef). Data are derived from a single experiment with 5 (mock, noHELP, uniHELP) or 10 (neoHELP) mice per group. Data in A-C were analyzed by a two-way ANOVA test followed by Tukey's multiple comparisons test. *p<0.05, **p<0.01, ***p<0.001, ****p<0.0001. (D) Fraction of $CD4^+$ T-cells expressing IL-2 and CD40L in response to tumor-specific neoantigens Ddr1, Pdch1 and Zmiz1. (E) Fraction of $CD4^+$ T-cells expressing IL-2 and CD40L in response to tumor-unrelated ‘universal’ antigens TTFC30, PADRE and HIV-nef58-68 (nef). (F) Fraction of $CD8^+$ T-cells expressing TNF and IFN-γ in response to tumor-specific neoantigens Irgq, Adpgk, Cpne1,Rpl18 and to an ovalbumin-derived reporter antigen (OVA). Data are derived from two independent experiments with 8 (mock, noHELP, uniHELP) or 13 (neoHELP) mice per group in total. Dots represent individual values, bars and whiskers represent means and standard errors (SEM), respectively. Data in D-F were analyzed by a Kruskal-Wallis test followed by Dunnet's multiple comparisons test. *p<0.05, **p<0.01, ***p<0.001, ****p<0.0001.

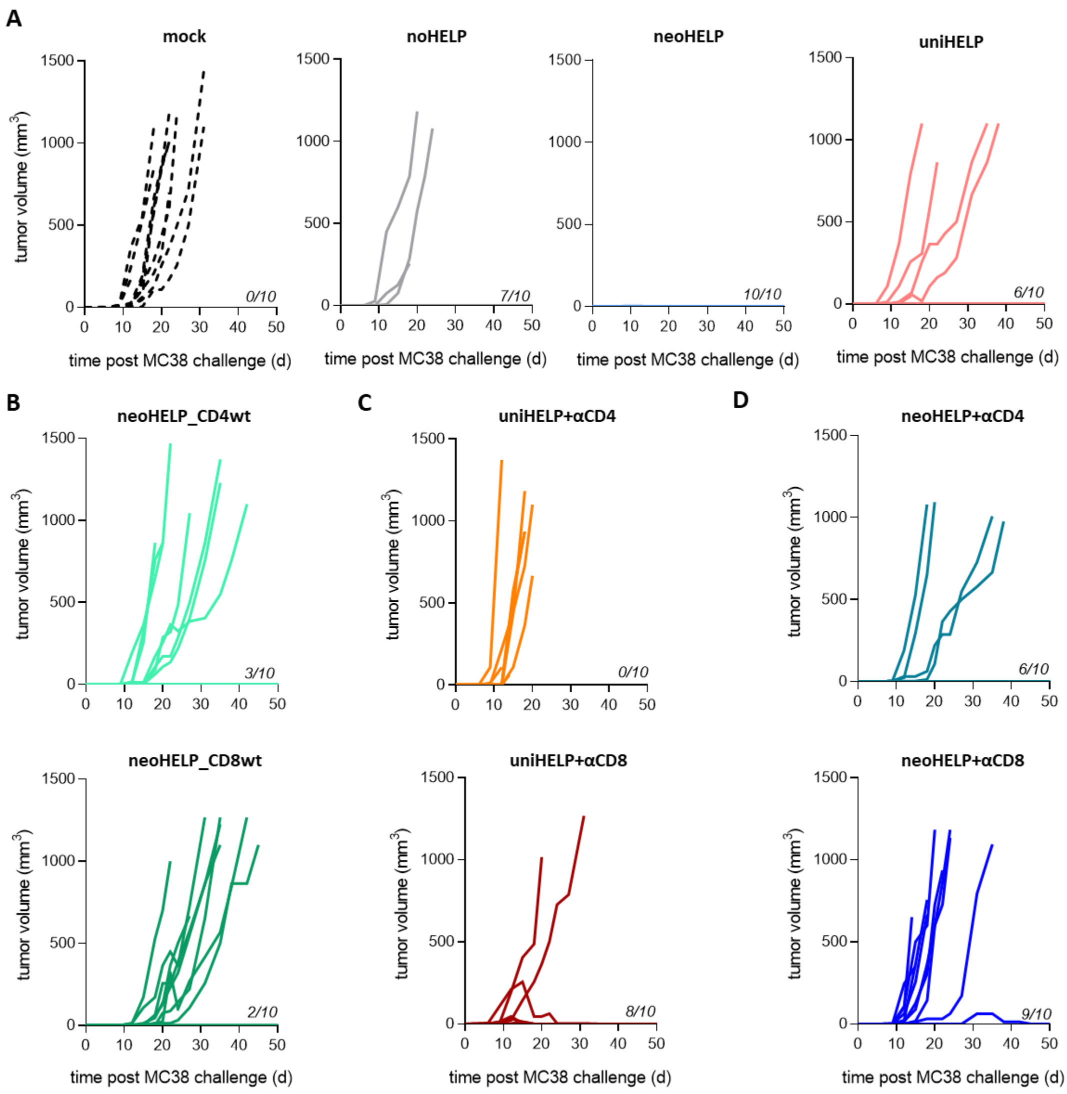

Figure S4. Tumor growth upon depletion of CD4 and CD8 responses. Mice (10 mice per group) were vaccinated three times, at 3-week intervals, with the indicated vaccines. Selected groups were depleted of $CD4^+$ or $CD8^+$ T-cells by three injections of 100 µg of either α-CD4 (clone GK1.5) or α-CD8 (clone 2.43) on days 57, 61 and 64 post primary vaccination. On day 63 post primary vaccination, mice were injected subcutaneously with 300.000 MC-38 colon carcinoma cells. Lengths and widths of the tumors were measured multiple times a week with a digital caliper to calculate tumor volume. Mice were removed from the experiment if the tumor exceeded 1000 mm^3 or if an ulcer occurred. Mice were vaccinated with (A) saline solution (mock), uniHELP or neoHELP (B) neoHELP vaccines carrying the wild-type counterparts of either the CD4 (neoHELP_CD4wt) or CD8 (neoHELP CD4wt) T-cell antigens. Additional groups of mice vaccinated with (C) uniHELP and (D) neoHELP underwent $CD4^+$ or $CD8^+$ T-cell depletion around the time of tumor challenge. Tumor volumes of individual mice are plotted, and the numbers of tumor-free mice at day 50 post challenge are indicated at the bottom right of each graph.

Table S1. CD8$^+$ and CD4$^+$ T-cell antigens used DNA vaccines

Antigen	Gene	Amino Acid (AA) sequence*	wt>mt (AA)	MHC-restriction
Irgq[43,46]	*Irgq*	QNAAKARDET**AALLNSAVL**GAAPLFVPPADCSSSD	G>V	H2-D^b
Adpgk[43–46,49]	*Adpgk*	DIPTGIPVHLEL**ASMTNMELM**SSIVHQQVFPTVAS	R>M	H2-D^b
Cpne1[43,46,49]	*Cpne1*	FTVGVDFTGSNGDP**SSPYSLHYL**SPTGVNEYLTAL	D>Y	H2-D^b
Rpl18[46,49]	*Rpl18*	RARSRILKAGG**KILTFDRL**ALESPKGRGTVLLSGP	Q>R	H2-D^b
OVA[67,68]	*SERPINB14*	LLPDEVSGLEQLE**SIINFEKL**TEWTSSNVMEERKI	n.a.	H2-K^b
Ddr2[48]	*Ddr2*	FKEVQCYFR**SEASEWEPHAVYFPLVLDDVNPS**ARF	T>H	I-A^b
Pcdh18[48]	*Pdhc18*	RFQRSRYEFVISENN**SPWAYITTVTATDPDL**GENG	G>W	I-A^b
Zmiz1[48]	*Zmiz1*	GIPPHT**RPPADFTQPAASAAAAA**VAAAAATATATA	A>S	I-A^b
TTFCp30[69,70]	*tetX*	**FNNFTVSFWLRVPKVSASHLE**	n.a.	I-A^b
PADRE[37]	*n.a.*	**AKFVAAWTLKAAA**	n.a.	I-A^b
HIV nef[38]	*nef*	**AWLEAQEEEEVGF**	n.a.	-

* T-cell epitopes in **boldface**, mutated residues underlined.

Table S2. Synthetic peptides used for intracellular cytokine staining (ICS) experiments

Antigen	Amino Acid (AA) sequence	Source
Irgq	RDETAALLNSAVLGAAPLFV	LUMC-Immunology synthetic peptide facility
Adpgk	ELASMTNMELMSSIV	LUMC-Immunology synthetic peptide facility
Cpne1	GSNGDPSSPYSLHYLSPTGVNE	LUMC-Immunology synthetic peptide facility
Rpl18	KAGGKILTFDRLALESPK	LUMC-Immunology synthetic peptide facility
OVA[67,68]	DEVSGLEQLESIINFEKLAAAAAK	LUMC-Immunology synthetic peptide facility
Ddr2	SEASEWEPHAVYFPLVLDDVNPS	LUMC-Immunology synthetic peptide facility
Pcdh18[48]	SPWAYITTVTATDPDL	LUMC-Immunology synthetic peptide facility
Zmiz1[48]	RPPADFTQPAASAAAAA	LUMC-Immunology synthetic peptide facility
TTFCp30	FNNFTVSFWLRVPKVSASHLE	LUMC-Immunology synthetic peptide facility
PADRE	AKFVAAWTLKAAA	LUMC-Immunology synthetic peptide facility
HIV nef	AWLEAQEEEEVGF	LUMC-Immunology synthetic peptide facility

Table S3. Flow cytometry antibody list

Antibodies	Fluorochrome	Source	Identifier
Fixable Viability Dye	eF450	Thermo Fisher	65-0863-14
Fixable Viability Dye	APC-eF780	Thermo Fisher	65-0865-14
CD3 (clone: 145-2C11)	BV510	BioLegend	100353
CD3 (clone: 145-2C11)	APC	Thermo Fisher	17-0031-83
CD4 (clone: RM4-5)	BV711	BioLegend	100550
CD4 (clone: GK1.5)	AF700	Thermo Fisher	56-0041-82
CD4 (clone: RM4-5)	PE-Cy7	Invitrogen	25-0042-82
CD8α (clone: 53-6.7)	APC-eF780	BioLegend	100744
CD8α (clone: 53-6.7)	APC-R700	BD Sciences	564983
CD8α (clone: 53-6.7)	BUV805	BD Horizon	612898
CD25 (clone: PC61)	BV711	BioLegend	102049
PD-1 (clone: 29F.1A12)	BV605	BioLegend	125225
LAG3 (clone: C9B7W)	PE-Cy7	Thermo Fisher	12-9942-81
CX3CR1 (clone: SA011F11)	BV785	Thermo Fisher	35-5893-82
KLRG1 (clone: 2F1)	FITC	BioLegend	138409
IL-2 (clone: JES6-5H4)	APC	BD Biosciences	554429
CD40L (clone: MR1)	PE	Thermo Fisher	12-1541-82
TNF (clone: MP6-XT22)	FITC	BioLegend	506304
IFNγ (clone: XMG1.2)	PE-Cy7	BD Biosciences	557649
H2-K^b/SIINFEKL	APC	LUMC-Immunology	tetramer facility
H2-K^b/Rpl18	APC	LUMC-Immunology	tetramer facility
H2-D^b/Adpgk	PE	LUMC-Immunology	tetramer facility
H2-D^b/Irgq	PE	LUMC-Immunology	tetramer facility

Table S4. Raw data of figures
