# Supplementary material for: Neoantigen-specific T cell help outperforms non-specific help in multi-antigen DNA vaccination against cancer
Source: Mol Ther Oncol. 2024 Jun 15;32(3):200835. doi: 10.1016/j.omton.2024.200835 (PMC11261851; doi:10.1016/j.omton.2024.200835)
Supplement: Document S2. Article plus supplemental information [file mmc3.pdf]

Molecular Therapy
Oncology
Original Article

Neoantigen-specific T cell help outperforms non-specific help in multi-antigen DNA vaccination against cancer

Joanna Fréderique de Graaf,[1,4] Tamara Pesic,[2,4] Felicia S. Spitzer,[2] Koen Oosterhuis,[1] Marcel G.M. Camps,[2] Iris Zoutendijk,[3] Bram Teunisse,[1] Wahwah Zhu,[3] Tsolere Arakelian,[2] Gerben C. Zondag,[1,3] Ramon Arens,[2] Jeroen van Bergen,[1,5] and Ferry Ossendorp[2,5]

[1]Immunetune BV, 2333 CH Leiden, the Netherlands; [2]Department of Immunology, Leiden University Medical Center, 2300 RC Leiden, the Netherlands; [3]Synvolux BV, 2333 CH Leiden, the Netherlands

CD4$^+$ T helper antigens are essential components of cancer vaccines, but the relevance of the source of these MHC class II-restricted antigens remains underexplored. To compare the effectiveness of tumor-specific versus tumor-unrelated helper antigens, we designed three DNA vaccines for the murine MC-38 colon carcinoma, encoding CD8$^+$ T cell neoantigens alone (noHELP) or in combination with either "universal" helper antigens (uniHELP) or helper neoantigens (neoHELP). Both types of helped vaccines increased the frequency of vaccine-induced CD8$^+$ T cells, and particularly uniHELP increased the fraction of KLRG1$^+$ and PD-1^{low} effector cells. However, when mice were subsequently injected with MC-38 cells, only neoHELP vaccination resulted in significantly better tumor control than noHELP. In contrast to uniHELP, neoHELP-induced tumor control was dependent on the presence of CD4$^+$ T cells, while both vaccines relied on CD8$^+$ T cells. In line with this, neoHELP variants containing wild-type counterparts of the CD4$^+$ or CD8$^+$ T cell neoantigens displayed reduced tumor control. These data indicate that optimal personalized cancer vaccines should include MHC class II-restricted neoantigens to elicit tumor-specific CD4$^+$ T cell help.

INTRODUCTION

Immune checkpoint blocking (ICB) therapy has revolutionized cancer therapy by eliciting curative responses in previously incurable types of cancer. In general, tumors with a high mutational burden (TMB) are more responsive to ICB therapy compared with patients with a low TMB.[1,2] A high TMB correlates with high frequencies of neoantigen-specific T cells, which recognize tumor-specific MHC-bound peptides resulting from these mutations.[3] Indeed, the presence of neoantigens and neoantigen-specific CD8$^+$ T cells correlates strongly with increased survival of ICB-treated patients.[4] These findings spurred the development of personalized cancer vaccines to elicit such beneficial neoantigen-specific T cell responses.[5–9]

Personalized neoantigen vaccines based on the prediction of MHC class I epitopes can broaden and expand pre-existent tumor-specific cytotoxic CD8$^+$ T cell responses, either as a stand-alone treatment or in conjunction with ICB therapy.[9–14] Such cancer vaccines employ a variety of technology platforms, including synthetic peptides, mRNA, and DNA.[5–9] All three types of vaccines are able to elicit effective anti-tumor CD8$^+$ T cell responses in mice,[6,7,15–17] and randomized controlled trials are beginning to show therapeutic efficacy in humans.[18] To prevent tumor immune escape and compensate for flawed neoantigen prediction algorithms, effective vaccines should include multiple antigens. Genetic vaccines are ideally suited for this, and mRNA- and DNA-based multi-antigen cancer vaccines currently under development typically contain 20 to 50 predicted neoantigens.[14,16,19,20]

CD8$^+$ T cell responses can be improved by CD4$^+$ T cell-mediated help, which is facilitated by different dendritic cells (DCs) in draining lymphoid tissue.[21,22] The primary helper signal is conferred via CD40L-CD40 signaling between CD4$^+$ T cells and DCs,[23–25] which is followed by cytokine secretion by the CD4$^+$ helper T cell toward the CD8$^+$ T cell.[26] The helped CD8$^+$ T cell response is characterized by downregulation of several immune regulatory markers, such as programmed cell death protein 1 (PD-1) and lymphocyte-activation gene 3 (LAG3), and upregulation of activation markers, including killer cell lectin-like receptor G1 (KLRG1), on antigen-specific CD8$^+$ T cells.[27,28] These helped CD8$^+$ T cells clear infections or tumors more efficiently compared with non-helped CD8$^+$ T cells and establish a durable memory response.[29–31] Accordingly, CD4$^+$ T cell responses are considered indispensable for an effective anti-tumor CD8$^+$ T cell response.

Several pre-clinical cancer vaccine studies confirmed that CD4$^+$ T cell help is required to induce effective and durable anti-tumor

Received 23 January 2024; accepted 13 June 2024;
https://doi.org/10.1016/j.omton.2024.200835.

[4]These authors contributed equally

[5]These authors contributed equally

Correspondence: Ferry Ossendorp, Department of Immunology, Leiden University Medical Center, 2300 RC Leiden, the Netherlands.
E-mail: f.a.ossendorp@lumc.nl

Published by Elsevier Inc. on behalf of The American Society of Gene and Cell Therapy.
This is an open access article under the CC BY license (http://creativecommons.org/licenses/by/4.0/).

responses,[6,7] even to MHC class II-negative tumors,[32] and that the presence of tumor-specific help enhances neoantigen-specific CD8$^+$ T cell responses.[33] In fact, a melanoma mRNA neoantigen vaccine originally designed to contain only CD8$^+$ T cell epitopes relied on unexpected vaccine-induced CD4$^+$ T cell responses for tumor eradication.[16] What's more, clinical studies indicated that neoantigen-specific CD4$^+$ T cells can be essential for the success of tumor-infiltrating lymphocyte therapy.[34,35] Thus, the inclusion of CD4$^+$ T cell antigens in human cancer vaccines is likely to improve their effectiveness.

The selection of vaccine neoantigens for CD4$^+$ T cells has proven challenging due to the lack of reliable algorithms to predict peptide binding to MHC class II.[36] To overcome this, universal, tumor-unrelated helper epitopes have been used instead.[28,37–41] Universal helper epitopes are selected based on their ability to bind to multiple MHC class II molecules and to elicit strong CD4$^+$ T cell responses. One of the best-characterized universal helper epitopes is PADRE (PAn-DR Epitope), a highly immunogenic peptide binding a broad panel of human MHC class II (HLA-DR) alleles.[37] Inclusion of universal CD4 epitopes in murine cancer vaccines resulted in improved effector functions of antigen-specific CD8$^+$ T cells and increased survival.[39,42] Due to the paucity of tumor-specific helper neoantigens identified in pre-clinical cancer models, it is currently unclear which type of CD4$^+$ T cell antigen most improves anti-tumor vaccine efficacy: tumor-specific neoantigens or tumor-unrelated universal helper antigens.

The murine MHC class II-negative MC-38 colon carcinoma resembles a typical clinical setting for neoantigen vaccination, as it carries a high TMB and neoantigen-specific T cell responses are present but strongly suppressed by the tumor microenvironment (TME). Several CD8$^+$ T cell epitopes have been identified by exome sequencing and mass spectrometric analysis of MHC class I-bound peptides.[8,43–47] Recently, we identified CD4$^+$ T cell neo-epitopes of this tumor by peptide elution from MC-38 cells induced to express MHC class II by transfection with the MHC class II transactivator (CIITA) gene.[48] Inclusion of these CD4$^+$ T cell peptides in a synthetic peptide vaccine containing CD8$^+$ T cell neo-epitopes improved survival of tumor-bearing mice.[48] Therefore, this model provided an unprecedented opportunity to compare the anti-tumor efficacy of tumor-unrelated and tumor-specific CD4$^+$ T cell help in neoantigen vaccination. In this study, we designed DNA vaccines encoding multiple CD8$^+$ T cell epitopes derived from the MC-38 colon carcinoma without (noHELP) or with universal- (uniHELP) or MC-38-derived (neoHELP) CD4$^+$ T cell epitopes, and tested their immunogenicity and tumor control.

RESULTS

Design of multi-antigen DNA vaccines noHELP, uniHELP, and neoHELP

The MC-38 model provided a unique opportunity to rigorously compare tumor-unrelated and tumor-specific help in neoantigen vaccination, as we and others recently identified CD8$^+$ and CD4$^+$ T cell neo-epitopes on this highly mutated, MHC class II-negative tumor cell line. First, mass spectrometric analysis of MHC class I-bound peptides from MC-38 cells identified a multitude of mutated peptides, including Adpgk, Cpne1, Irgq, and Rpl18,[43,46,49] while MHC class II elution experiments from CIITA-expressing MC-38 cells found seven mutated peptides, including Zmiz1, Pcdh18, and Ddr2.[48] Importantly, no Zmiz1, Pcdh18, or Ddr2 peptides were detected in the MHC class I eluate of MC-38, and no Adpgk, Cpne1, Irgq, or Rpl18 peptides were found in the class II molecules of MC-38-CIITA.[46,48] Second, CD8$^+$- and not CD4$^+$-T cell responses to Adpgk, Cpne1, Irgq, and Rpl18 have been reported in response to vaccination with peptide (Adpgk, Irgq, Rpl18),[45–47] DNA (Adpgk, Cpne1, Irgq),[19,44] or irradiated MC-38 cells (Adpgk, Rpl18).[46] Conversely, vaccination with synthetic peptides generated CD4$^+$, not CD8$^+$, T cell responses to Zmiz1, Pcdh18, and Ddr2.[46] Finally, while therapeutic vaccination using mutated Adpgk or Rpl18 peptides already reduced MC-38 tumor growth,[43,46] therapeutic efficacy was further improved when these MHC class I-binding peptides were combined with MHC class II-binding peptides Zmiz1, Pcdh18, and Ddr2.[48] This allowed the design of multi-neoantigen vaccines inducing CD8$^+$ T cell responses in the absence or presence of CD4$^+$ T cell responses.

Three DNA vaccines were designed using the neo-epitopes mentioned above (Figure 1A). All constructs coded for four CD8$^+$ T cell neo-epitopes, derived from Irgq, Adpgk, Cpne1, and Rpl18, and CD8$^+$ T cell reporter epitope $OVA_{257-264}$ (OVA, derived from ovalbumin). To allow correct processing of the epitopes from their natural context, their sequences were extended at both ends by their naturally flanking amino acids and separated by short spacers, thus creating multi-antigen vaccines (see materials and methods). The control "noHELP" DNA vaccine contained only these CD8$^+$ T cell antigens (Figure 1A). In addition to the CD8$^+$ T cell antigens, the neoHELP vaccine included the three immunogenic CD4$^+$ T cell neoantigens recently identified by our group, derived from Ddr2, Pcdh18, and Zmiz1.[48] The uniHELP vaccine contained the same CD8$^+$ T cell antigens combined with three universal, non-tumor-related CD4$^+$ T cell antigens, including PADRE as well as TTFCp30-and HIV-nef-derived antigens.[37–39] Cells transfected with noHELP, neoHELP, or uniHELP plasmids expressed similar amounts of multi-antigen proteins of the expected sizes, and were recognized equally well by a T cell line specific for the C-terminal OVA reporter epitope, thus confirming correct expression and processing of the multi-antigen proteins (Figure S1). The plasmids were used as templates for the production of synthetic, linear DNA vaccines.

DNA vaccines noHELP, uniHELP, and neoHELP induce functional CD4$^+$ and CD8$^+$ T cell responses

To determine whether the vaccines induced the expected CD4$^+$ and CD8$^+$ T cell specificities *in vivo*, C57BL/6 mice were vaccinated intradermally with the linear DNA vaccines, or mock-vaccinated. After three vaccinations, spleen cells were stimulated *ex vivo* with synthetic peptide versions of the vaccine antigens, and T cell responses were evaluated by intracellular cytokine staining (ICS) (Figures 1, S3A, and S3B). Splenic CD8$^+$, but not CD4$^+$, T cells from mice vaccinated

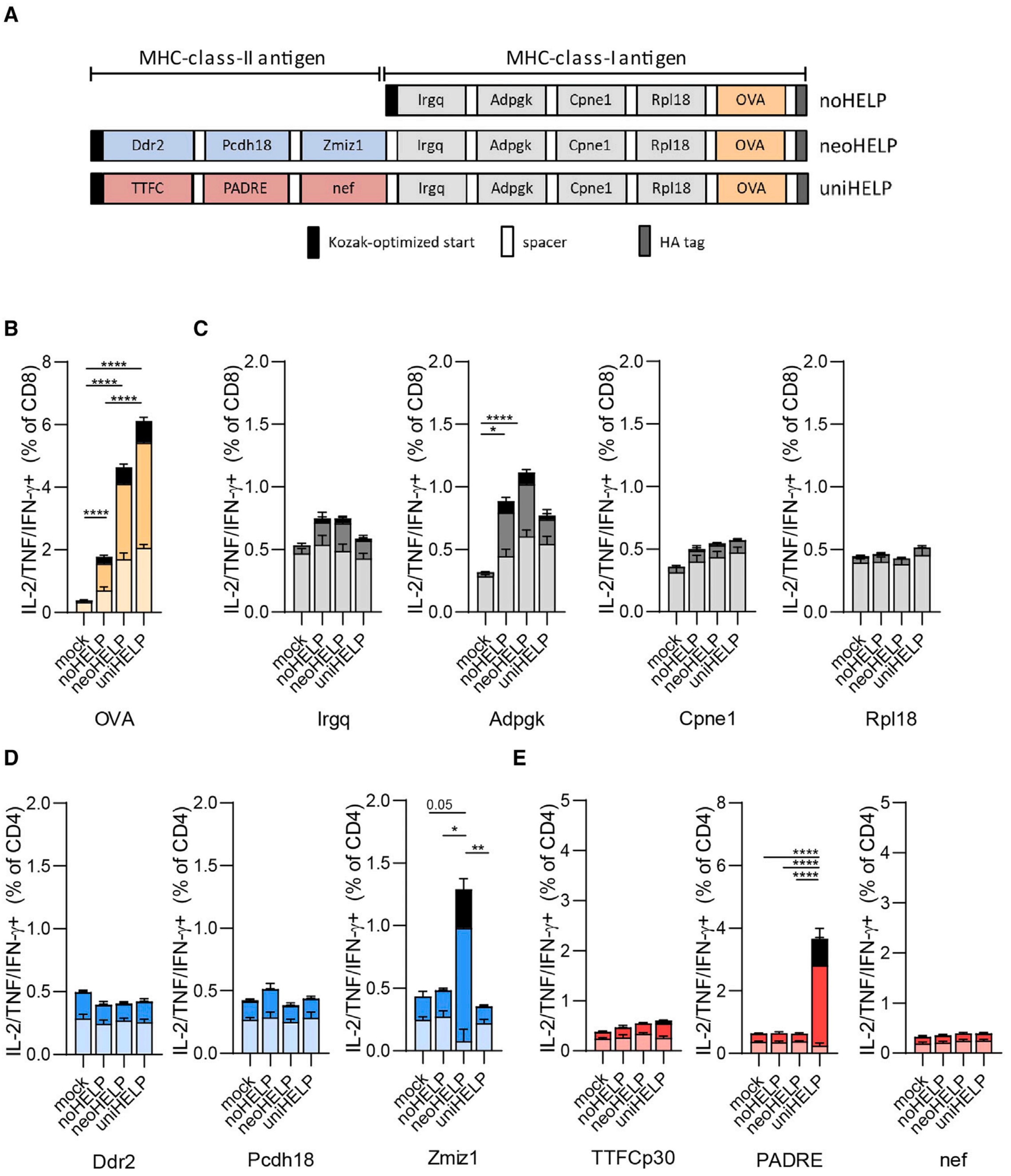

Figure 1. Multi-antigen DNA vaccines induce functional T cell responses against MHC class I- and MHC class II-restricted (neo)antigens

(A) Schematic representation of the coding sequences of multi-neoantigen vaccines noHELP, uniHELP, and neoHELP. (B–E) Mice were vaccinated with the indicated vaccines three times, at 3-week intervals. Ten days after the final vaccination, spleen cells were cultured with dendritic cells loaded with indicated peptides for 5 h, and analyzed by intracellular cytokine staining (ICS). IL-2-, TNF-, and/or IFN-γ-positive $CD4^+$ or $CD8^+$ T cells upon stimulation with the indicated individual long synthetic peptides: (B) OVA24, (C) Irgq, Adpgk, Cpne1, Rpl18,[43,46] (D) Ddr2, Pcdh18, Zmiz1,[48] (E) TTFCp30, PADRE, HIV-nef58-68 (nef).[39] Colors indicate T cells secreting a single cytokine (pastel color), two cytokines (dark color), or three cytokines (black). Data are derived from a single experiment with 5 (mock, noHELP, uniHELP) or 10 (neoHELP) mice per group, representative of 2 independent experiments (shown in Figures S3D–S3F). Dots represent individual values, bars and whiskers represent means and standard errors (SEM), respectively. Data were analyzed by a two-way ANOVA test followed by Tukey's multiple comparisons test. *$p < 0.05$, **$p < 0.01$, ***$p < 0.001$, ****$p < 0.0001$.

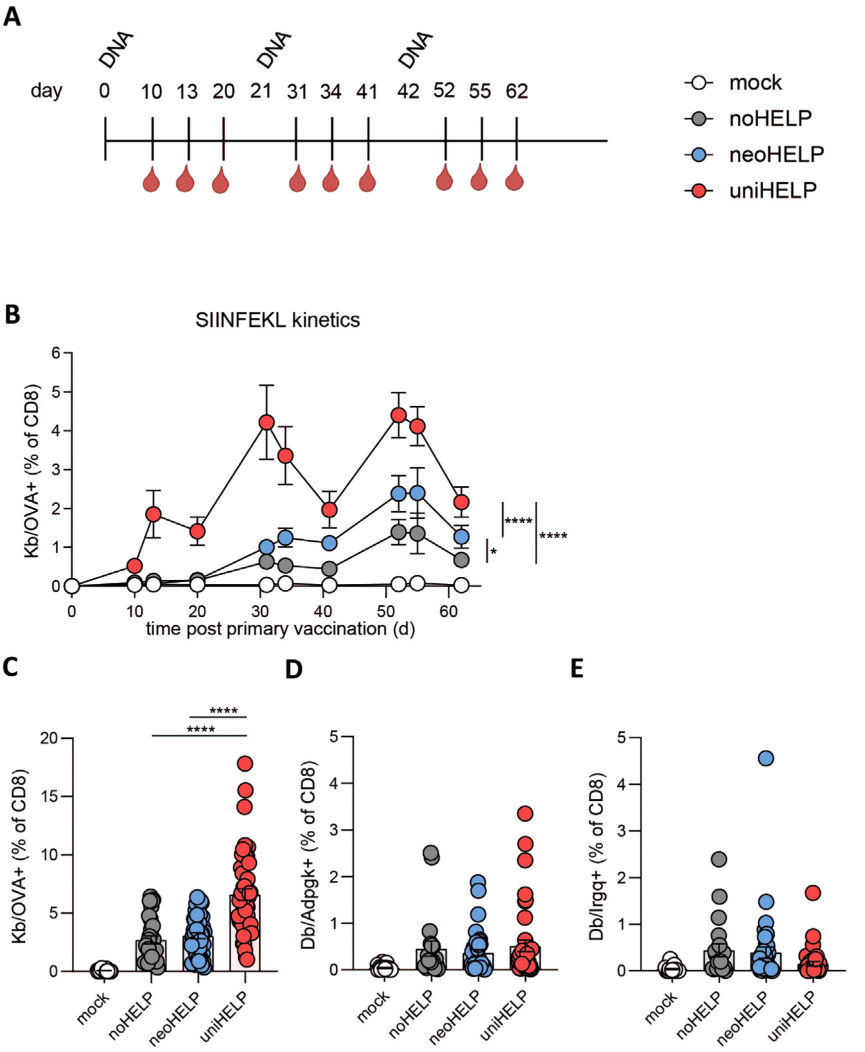

Figure 2. CD4 helper antigens increase the frequency of vaccine-induced CD8⁺ T cells in blood

(A) Schematic overview of the experiment: mice were vaccinated intradermally three times, at 3-week intervals, with the indicated vaccines. Mice were bled at the indicated time points to quantify antigen-specific $CD8^+$ T cells by flow cytometry (Figures S2A and S2B). (B) Kinetics of OVA-specific $CD8^+$ T cell responses in blood. Data are derived from a single experiment with 10 mice per group, representative of 3 experiments. Bars and whiskers represent means and standard errors (SEM), respectively. Data were analyzed in mixed-effects analysis followed by Tukey's multiple comparisons test comparing noHELP, neoHELP, and uniHELP. **p* < 0.05, ***p* < 0.01, ****p* < 0.001, *****p* < 0.0001. (C) OVA-, (D) Adpgk-, (E) Irgq-specific $CD8^+$ T cell percentages in blood at the peak of the tertiary response, 52 days post primary vaccination. Data in (C)–(E) are from 3 independent experiments with 50 mice per group in total. Dots represent individual values, while bars and whiskers represent means and standard errors (SEM). Data were analyzed by one-way ANOVA followed by Tukey's multiple comparisons test comparing noHELP, neoHELP, and uniHELP. **p* < 0.05, ***p* < 0.01, ****p* < 0.001, *****p* < 0.0001.

with noHELP, neoHELP, and uniHELP produced IL-2, TNF, and/or IFN-γ in response to a pool of the CD8 neoantigen peptides (Figure S3A). Importantly, only $CD4^+$ T cells from neoHELP-vaccinated mice produced these cytokines in response to a peptide pool containing the MHC class II-presented neoantigens, and only $CD4^+$ T cells from uniHELP-vaccinated mice responded to the uniHELP peptide pool (Figures S3B and S3C). Stimulation with individual peptides revealed that $CD8^+$ T cells specifically recognized OVA and Adpgk, weakly recognized Irgq, but did not respond to Cpne1 or Rpl18 (Figures 1B, 1C, and S3F). Of note, compared with noHELP, neoHELP, and uniHELP induced more single, double, and triple cytokine-producing OVA-specific $CD8^+$ T cells. $CD4^+$ T cells from neoHELP-vaccinated mice produced IL-2, TNF, and/or IFN-γ in response to Zmiz1, but not to Pcdh18 or Ddr2 peptides (Figures 1D and S3D), whereas in uniHELP-vaccinated mice, only PADRE generated $CD4^+$ T cell cytokine responses (Figures 1E and S3E). In summary, while all three vaccines induced functional $CD8^+$ T cell responses against the OVA, Adpgk, and Irgq antigens, neoHELP generated a tumor-specific $CD4^+$ T cell response against MHC class II-restricted neoantigen Zmiz1, and uniHELP induced a tumor-unrelated $CD4^+$ T cell response to universal helper antigen PADRE.

Inclusion of CD4 helper antigens increases the frequency of vaccine-induced CD8⁺ T cells

Having shown that both helper cassettes induce $CD4^+$ T cell responses to at least one of the encoded antigens, we next determined the effect of tumor-unrelated and tumor-related $CD4^+$ T cell help on the induction of $CD8^+$ T cell responses. MHC-peptide tetramers were used to quantify antigen-specific $CD8^+$ T cells in the blood of mock-, noHELP-, neoHELP-, and uniHELP-vaccinated mice (Figure 2A). All three vaccines generated detectable levels of OVA-specific $CD8^+$ T cells, which—in line with the splenic responses (Figure 1B)—were significantly increased in neoHELP- and uniHELP-vaccinated mice compared with noHELP-vaccinated mice (Figure 2B). Throughout the priming and expansion phase, uniHELP induced an even more pronounced OVA-specific $CD8^+$ T cell response than neoHELP (Figure 2B), an effect that was maintained after three vaccinations (Figure 2C). Neoantigen-specific $CD8^+$ T cells were also detectable (Figures 2D and 2E), but their levels were too low to reveal helper effects. In summary, the addition of both tumor-unrelated and tumor-related CD4 helper antigens increased vaccine-induced $CD8^+$ T cell frequencies in the circulation, but the tumor-unrelated universal helper antigens were

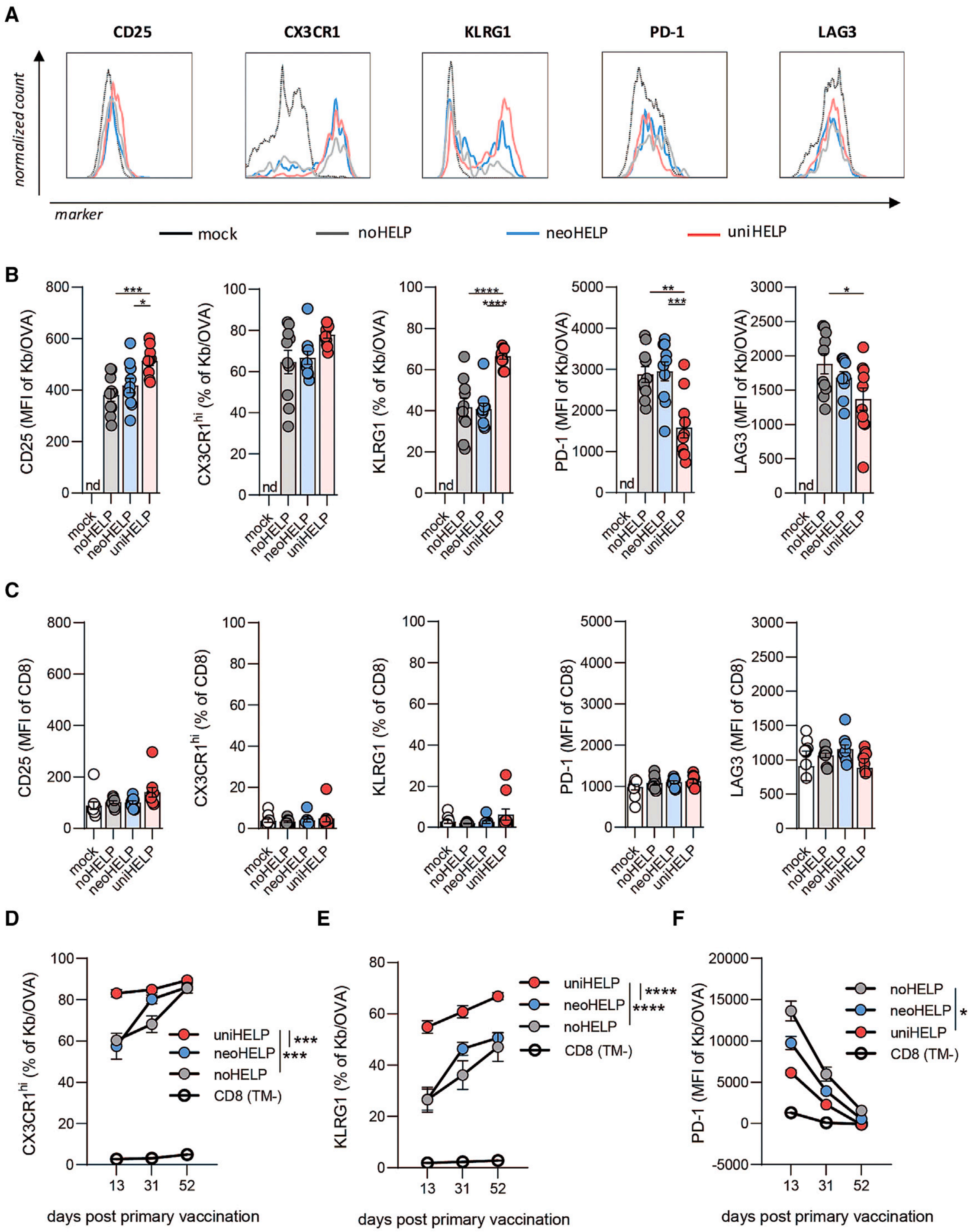

Figure 3. CD4 helper antigens promote phenotypic changes in vaccine-induced CD8$^+$ T cells associated with improved effector function

Mice were vaccinated intradermally three times, at 3-week intervals as indicated in Figure 2. Mice were bled at the peak of the T cell response following the third vaccination (day 52), after which expression of the indicated markers on OVA-specific CD8$^+$ T cells was measured by flow cytometry. (A) Histograms show normalized counts of cells expressing surface markers CD25, CX3CR1, KLRG1, PD-1, and LAG3 on total CD8$^+$ T cells of mock-vaccinated mice or OVA-specific CD8$^+$ T cells, identified by Kb/OVA-tetramer staining, in mice vaccinated with noHELP, neoHELP, or uniHELP (Figures S2C and S2D). Marker expression on (B) OVA-specific CD8$^+$ T cells or (C) tetramer-negative CD8$^+$ T cells of mock-, noHELP-, neoHELP-, or uniHELP-vaccinated mice. Expression was quantified as mean fluorescence intensity (MFI) or percentage of marker-positive cells, as indicated. Dots represent individual values, bars and whiskers represent mean and standard error (SEM). Data were derived from a single experiment,

(legend continued on next page)

(uniHELP) more potent in this respect than the tumor-specific helper neoantigens (neoHELP).

Inclusion of CD4 helper antigens results in phenotypic changes in vaccine-induced CD8$^+$ T cells associated with improved effector function

Adequate CD4$^+$ T cell help leads to increased CD8$^+$ T cell functionality, which is associated with elevated levels of activation and differentiation markers (e.g., CD25, KLRG1, CX3CR1) as well as downregulation of several co-inhibitory receptors (e.g., PD-1, LAG3).[27,28] We therefore explored the impact of the different types of helper antigens on the phenotype of antigen-specific CD8$^+$ T cells (Figure 3).

After three vaccinations with noHELP, neoHELP, or uniHELP, surface expression of CD25, CX3CR1, and KLRG1 was elevated on circulating OVA-specific CD8$^+$ T cells (Figure 3B) compared with non-OVA-specific CD8$^+$ T cells (Figure 3C). UniHELP vaccination further increased expression levels of CD25, KLRG1, and CX3CR1 on OVA-specific CD8$^+$ T cells compared with noHELP vaccination, while no significant effect of neoHELP on these markers was observed. Checkpoint inhibitor receptors PD-1 and LAG3 were also generally elevated on OVA-specific versus non-OVA-specific CD8$^+$ T cells (Figures 3B and 3C). Relative to noHELP, in this case uniHELP caused significant downregulation of these receptors on OVA-specific CD8$^+$ T cells, while the effect of neoHELP was limited to LAG3 and did not reach statistical significance (Figure 3B). Thus, the inclusion of tumor-unrelated universal CD4 helper antigens (uniHELP) resulted in a clear "helper signature" on vaccine-induced CD8$^+$ T cells, while this signature was marginally detectable in the case of tumor-specific helper neoantigens (neoHELP).

The expression of these markers on vaccine-induced CD8$^+$ T cells changed gradually over time, resulting in a more "helped" phenotype of CD8$^+$ T cells with each vaccination (Figures 3D–3F). Again, compared with noHELP, uniHELP vaccination resulted in significantly greater fractions of antigen-specific CD8$^+$ T cells expressing CX3CR1 at high levels (Figure 3D) and KLRG1 (Figure 3E). Again, this pattern was reversed for PD-1 (Figure 3F). In particular after the second vaccination, the helper signature in the neoHELP group was present but less pronounced than in the uniHELP group (Figures 3D–3F). Together, these data demonstrated that vaccination with constructs containing CD4 helper antigens, most prominently with tumor-unrelated universal helper antigens (uniHELP), resulted in phenotypic changes in vaccine-induced CD8$^+$ T cells that have been associated with improved effector functions.[27,50]

Tumor-specific, but not tumor-unrelated, CD4 helper vaccine antigens improve tumor control

As the inclusion of CD4 helper antigens improved CD8$^+$ T cell responses both quantitatively and qualitatively, we compared the vaccines for their ability to induce protective anti-tumor immunity. Three weeks after the third vaccination, mice were injected subcutaneously with wild-type, MHC class II-negative, MC-38 tumor cells. Within 5 days, tumors started to grow in all control, mock-vaccinated mice, and within a month all mice succumbed to their tumor (Figures 4A and 4B). The same progressive tumor growth was observed in 85% of noHELP-vaccinated mice (Figure 4A), although a small but significant minority (15%) did not develop a tumor and survived (Figure 4B). The addition of universal helper antigens to the neoantigen vaccine did not improve upon this: even though 15% of the mice in the uniHELP group displayed delayed tumor growth (Figure 4A), just 15% ultimately survived tumor-free (Figure 4B). In stark contrast, only 30% of the mice that had received neoHELP developed a tumor, and tumor growth was often delayed compared with mock- and noHELP-vaccinated mice (Figure 4A). What's more, 70% of neoHELP group did not develop a palpable tumor and survived long term, a significant improvement compared with the other experimental groups (Figure 4B). In short, including tumor-derived helper neoantigens, but not tumor-unrelated universal antigens, in a neoantigen vaccine greatly enhanced immune control of MC-38 tumors.

Neoantigen-specific CD4$^+$ and CD8$^+$ T cells both contribute to tumor control

NeoHELP, the vaccine containing both MHC class I- and MHC class II-restricted neoantigens, best protected against a lethal tumor challenge. To assess the relative importance of each of these neoantigen categories, we designed additional neoHELP constructs (Figure 5A) containing the wild-type (wt) counterparts of either the class I-restricted CD8$^+$ T cell antigens (neoHELP_CD8wt) or the class II-restricted CD4$^+$ T cell antigens (neoHELP_CD4wt). In these DNA vaccines, the mutated amino acids responsible for neoantigen formation were reverted to the wild-type amino acids, and the resulting sequences were therefore immunologically self (see Table S1). The vaccinated mice were challenged with a lethal dose of MC-38 tumor cells 3 weeks after the third and final DNA vaccination. In line with our earlier results (Figure 4), vaccination with neoHELP resulted in 100% tumor control, significantly better than the uniHELP and noHELP vaccines that protected 60%–70% of the mice (Figure 5B). Compared with neoHELP, vaccination with neoHELP_CD8wt or neoHELP_CD4wt significantly reduced survival from 100% to 20%–30% (Figure 5C), revealing that both MHC class I- and class II-restricted neoantigens were crucial for tumor control.

representative of three experiments, with 10 mice per group. Data were analyzed by one-way ANOVA followed by Tukey's multiple comparisons test comparing noHELP, neoHELP, and uniHELP. $^*p < 0.05$, $^{**}p < 0.01$, $^{***}p < 0.001$, $^{****}p < 0.0001$. (D) CX3CR1^{hi}, (E) KLRG1, or (F) PD-1 expression on OVA-specific CD8$^+$ T cells monitored in blood over time on days 13, 31, and 52 post primary vaccination. Data are derived from a single experiment, representative of three, with 10 (noHELP) or 30 (neoHELP and uniHELP) mice per group. Data from tetramer-negative (TM$^-$) CD8$^+$ T cells from all experimental groups were pooled and plotted as CD8 as background reference. Dots and whiskers represent means and standard errors (SEM), respectively. Data were tested in mixed-effects analysis followed by Tukey's multiple comparisons test comparing noHELP, neoHELP, and uniHELP. $^*p < 0.05$, $^{**}p < 0.01$, $^{***}p < 0.001$, $^{****}p < 0.0001$. nd, no OVA-specific T cells detected.

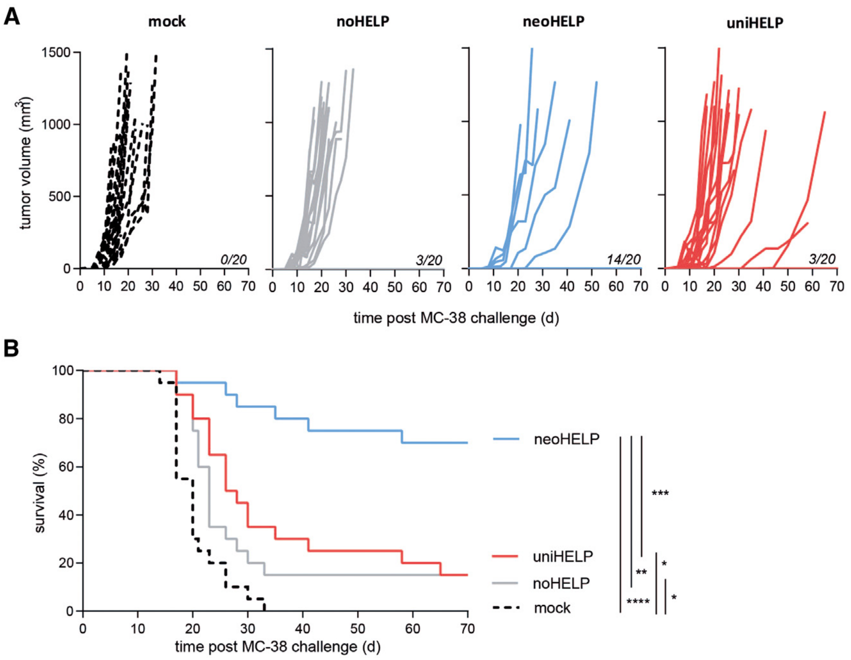

Figure 4. Tumor-derived, but not tumor-unrelated, CD4 helper antigens improve tumor control

Mice were vaccinated intradermally with the indicated vaccines three times, at 3-week intervals, followed by subcutaneous injection of MC-38 colon carcinoma cells after another 3 weeks. (A) Tumor volumes of individual mice were tracked over time. Mice were euthanized when volumes exceeded 1,000 mm^3. Numbers of tumor-free mice at day 70 post challenge are indicated. (B) Survival of mice was plotted over time. Data are derived from 2 independent experiments with 20 mice per experimental group in total. Statistical significance was determined using the Gehan-Breslow-Wilcoxon test. $^{*}p < 0.05$, $^{**}p < 0.01$, $^{***}p < 0.001$, $^{****}p < 0.0001$.

To examine whether vaccine-induced tumor protection was dependent on $CD4^+$ or $CD8^+$ T cells in the effector phase, we performed *in vivo* depletion of these two T cell subsets in uniHELP- and neoHELP-immunized mice around the time of MC-38 inoculation (Figures 5D and 5E). While $CD4^+$ T cell depletion did not significantly affect survival of uniHELP-vaccinated mice (Figure 5D), it markedly reduced survival of the neoHELP group from 100% to 60% (Figure 5E). In both groups, removal of $CD8^+$ T cells (almost) completely abrogated tumor control (Figures 5D and 5E). Together, these findings indicate a crucial role for vaccine-elicited $CD4^+$ T cells recognizing tumor-specific neoantigens, but not tumor-unrelated universal antigens, in optimal immune control of the MC-38 tumor.

DISCUSSION

In this study, we used the MHC class II-negative murine colon carcinoma MC-38 to compare tumor-specific and tumor-unrelated $CD4^+$ T cell helper antigens in neoantigen vaccination. Three multi-antigen DNA vaccines were designed that induced neoantigen-specific $CD8^+$ T cell responses only (noHELP) or together with $CD4^+$ T cell responses to either tumor-unrelated “universal” antigens (uniHELP) or MC-38-derived neoantigens (neoHELP). Compared with noHELP, neoHELP and uniHELP improved vaccine-induced $CD8^+$ T cell responses both quantitatively and qualitatively. Although this improvement was most pronounced in the case of uniHELP, only neoHELP vaccination increased protection against outgrowth of the MC-38 colon carcinoma. NeoHELP-induced tumor control was dependent on the presence of $CD4^+$ and $CD8^+$ T cells, and neoHELP variants encoding the non-mutated, wild-type counterparts of either the CD4 or CD8 neoantigens caused reduced tumor control. These data indicate that, for optimal effectiveness, personalized cancer vaccines should contain tumor-specific rather than tumor-unrelated $CD4^+$ T cell antigens.

The inclusion of tumor-unrelated and tumor-specific $CD4^+$ T cell helper antigens in the neoantigen vaccines increased not only the frequency of vaccine-induced $CD8^+$ T cells, but also altered their surface phenotype. In the presence of $CD4^+$ T cell help, these $CD8^+$ T cells were more often $KLRG1^+$ and had lower PD-1 expression. This helper signature on the $CD8^+$ T cells, which is associated with improved effector function of memory T cells[51] and increased tissue infiltration,[27,52] was most pronounced in the case of tumor-unrelated universal helper antigens. At the time of tumor injection, therefore, uniHELP-vaccinated mice most likely had more and better tumor neoantigen-specific $CD8^+$ T cells than the other groups. Non-helped neoantigen-specific $CD8^+$ T cells already had some effect on tumor resistance, since tumor-free survival was significantly greater in the noHELP group compared with mock-vaccinated mice. However, since uniHELP-vaccinated mice fared no better than noHELP-vaccinated mice, the quantity and quality of these $CD8^+$ T cells had no discernable effect on protection in this setting. The same universal helper cassette did improve protection by an HPV16-E7-based DNA vaccine against outgrowth of the E7-containing TC-1 line,[39] suggesting that vaccine efficacy in some cases does benefit from highly immunogenic tumor-unrelated helper antigens.

In contrast to tumor-unrelated helper antigens, tumor-related helper neoantigens did significantly improve tumor resistance. This was unlikely to be the result of improved neoantigen-specific $CD8^+$ T cell responses before tumor challenge, as we observed fewer tetramer-positive and cytokine-producing $CD8^+$ T cells in blood and spleen. Strongly reduced tumor resistance of neoHELP-vaccinated mice caused by $CD4^+$ T cell depletion around the time of MC-38 inoculation indicated that neoantigen-specific $CD4^+$ T cells played a major role in the effector phase of the anti-tumor response. Upon subcutaneous injection of MC-38 cells, tumor antigens can be taken up by DCs and presented to $CD4^+$ and $CD8^+$ T cells in tumor-draining lymph nodes. This provides an opportunity for vaccine-induced

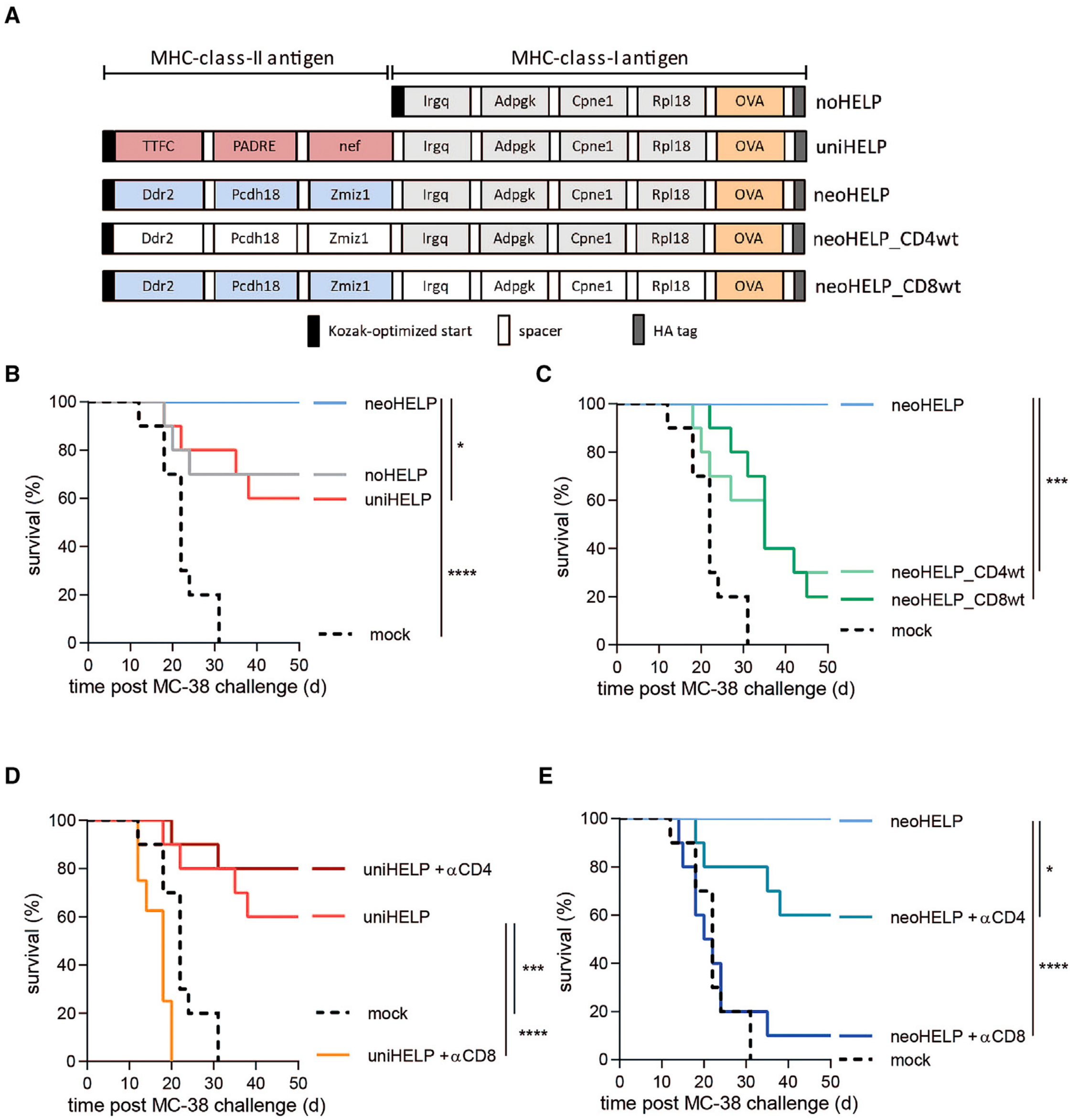

Figure 5. Neoantigen-specific CD4$^+$ and CD8$^+$ T cells both contribute to tumor control

(A) Schematic representation of the coding sequences of the neoantigen vaccines. Wild-type counterparts of neoantigen sequences in neoHELP_CD4wt and neoHELP_CD8wt are indicated in white. Mice (10 per group) were injected three times, at 3-week intervals, with these vaccines. Three weeks after the final vaccination (day 63), mice were injected subcutaneously with MC-38 colon carcinoma cells. Additional neoHELP and uniHELP groups were depleted of CD4$^+$ or CD8$^+$ T cells by intraperitoneal injection of depleting antibodies around the time of tumor challenge (days 57, 61, and 64). Tumor size was tracked (Figure S4) and mice were euthanized if it exceeded 1,000 mm^3. (B–E) For clarity, survival is shown in four separate panels, depicting mice vaccinated with (B) mock, uniHELP, neoHELP, (C) mock, neoHELP, neoHELP_CD4wt, neoHELP_CD8wt, (D) mock, uniHELP, uniHELP + CD4$^+$ T cell depletion, uniHELP + CD8$^+$ T cell depletion, or (E) mock, neoHELP, neoHELP + CD4$^+$ T cell depletion, neoHELP + CD8$^+$ T cell depletion. Statistical significance was determined using the Gehan-Breslow-Wilcoxon test. *$p < 0.05$, **$p < 0.01$, ***$p < 0.001$, ****$p < 0.0001$.

neoantigen-specific CD4$^+$ T cells to provide optimal help and stimulate tumor-specific CD8$^+$ T cells, which can then re-enter the circulation and home to the tumors to kill malignant cells.[53] In addition to their role in the tumor-draining lymph nodes, tumor-specific CD4$^+$ T cells residing in the TME can contribute to tumor resistance, for example, by recruiting[54–56] and providing local help to[57] CD8$^+$ T cells. Which of these mechanisms contributed to the efficacy of the neoHELP vaccine is at present unclear and will be the subject of future studies.

Irrespective of the exact immunological mechanisms, several studies have shown that the inclusion of either tumor-specific[32,33,58,59] or

tumor-unrelated[27,39,59,60] helper antigens can improve the effectiveness of cancer vaccines. For example, the universal helper cassette used in this study did improve protection by an HPV16-E7-based DNA vaccine against outgrowth of the E7-containing TC-1 line.[39] However, in specific settings the inclusion of a universal helper antigen in a vaccine can in fact reduce anti-tumor CD8$^+$ T cell immunity, most likely by interfering with vaccine-induced tumor-specific CD4$^+$ T cell responses.[61] In addition to our study, we are aware of only a single other study that performed a head-to-head comparison of the two classes of helper antigens.[58] Dolina et al. recently discovered several CD4$^+$ and CD8$^+$ T cell epitopes on an aggressive low-TMB squamous cell tumor cell line.[58] In their hands, synthetic peptide vaccines consisting of a minimal CD8$^+$ T cell epitope and either a CD4$^+$ T cell neoepitope or universal helper peptide PADRE protected equally well against a subsequent tumor challenge.[59] On balance, tumor-specific helper antigens appear to perform at least as well or considerably better than highly immunogenic tumor-unrelated helper antigens, indicating that they should be the preferred choice for inclusion in vaccines.

Together with several other studies,[16,33,58,59] our experiments demonstrate the value of MHC class II-restricted tumor-specific antigens in cancer vaccines. As the MHC class II peptide-binding motifs are far less restrictive than those for MHC class I, the prediction algorithms for MHC class II epitopes are less well developed.[62] However, using a large dataset of HLA-DR-binding peptides identified by peptide elution and mass spectrometry to train their prediction algorithm, Attermann et al. were recently able to achieve prediction accuracies for HLA-DR comparable with those previously reserved for HLA-A and HLA-B.[62] As MHC class II prediction methods continue to be optimized, for example, by using highly sensitive molecular identification methods employing CIITA to drive MHC II expression on patient-derived cancer cells,[48,63] the incorporation of CD4$^+$ T cell antigens into personalized cancer vaccines should become feasible.[64–67] Our data demonstrate that multi-antigen DNA vaccines, previously reported to induce preferentially CD8$^+$ T cell responses,[17] can be excellent inducers of functional CD4$^+$ T cell responses.

In summary, we show that including tumor-specific, but not tumor-unrelated, MHC class II-restricted CD4$^+$ T cell antigens in a cancer vaccine resulted in increased control of tumor growth. Thus, our data emphasize the need to include tumor-specific MHC class II-restricted antigens in personalized neoantigen vaccines, as well as the need for improved identification tools for such antigens.

MATERIALS AND METHODS

Cell lines

The murine MC-38-L cell line, referred to as MC-38, was used in all experiments.[43,48] MC-38 was cultured in Iscove's modified Dulbecco's medium (IMDM) (Capricorn Scientific, Frankfurt, Germany) supplemented with 8% fetal calf serum (Sigma-Aldrich, St. Louis, MO), 50 μg/mL streptomycin (Gibco, Carlsbad, CA), 50 IU/mL penicillin (Gibco), 2 mM L-glutamine (Gibco), and 30 μM β-mercaptoethanol (Merck Millipore, Kenilworth, NJ), in a humidified CO_2 incubator (37°C, 5% CO_2). The cell lines B3Z B16-F10 and HEK293T were cultured in supplemented IMDM, but without β-mercaptoethanol. The immature DC cell line, D1, was cultured in IMDM medium supplemented with 30% supernatant of the R1 cell line expressing granulocyte-macrophage colony-stimulating factor. All cell lines were regularly checked for mycoplasma infection and found to be negative.

Mice

Female C57BL/6J mice (6–8 weeks old) were purchased from Janvier Labs (Le Genest Saint Isle, France). Mice were housed under specific pathogen-free conditions in individually ventilated cages at the Leiden University Medical Center (LUMC) animal facility. All animal experiments were performed in accordance with Dutch Animal Ethical Committee guidelines and were approved by the Animal Welfare body of LUMC (DEC consult number: AVD11600202013796).

DNA vaccines

Codon-optimized DNA constructs encoding multiple CD4$^+$ and CD8$^+$ T cell antigens (Table S1) were first produced as plasmids by Gibson assembly,[8] and then used as templates for the synthesis of linear DNA vaccines.[68] In short, this cell-free method relies on primer-free, isothermal, rolling-circle amplification (RCA) using a high-fidelity DNA polymerase with strand-displacement activity. However, instead of using oligonucleotides to prime the RCA reaction, RNA polymerase is combined with ribonucleotides to generate a small RNA "primer." After the RCA reaction, the expression cassette, containing the coding sequence as well as regulatory sequences (promoter, poly-adenylation signal), is excised. Subsequent capping of the expression cassette with oligonucleotides renders this cassette, but not the plasmid backbone, resistant to exonucleases. I*n vivo* expression and immunogenicity of synthetic linear DNA vaccines produced in this manner are equivalent in magnitude and kinetics to equimolar amounts of the corresponding plasmid DNA vaccines (data not shown as has been reported for linear DNA vaccines produced using a different method.[69] For the experiments described here, the vaccines underwent two purification steps on a Macherey-Nagel Nucleobond column (Dueren, Germany), followed by centrifugation (30 min, 10,000 × *g*, 4°C) to remove any remaining debris.

Five multi-antigen linear DNA vaccines were produced (Figures 1A and 5A). The base construct (noHELP) coded for five 35-mer CD8$^+$ T cell antigens separated by a triple alanine linker (AAA): MC-38 neoantigens Irgq, Adpgk, Cpne1, Rpl18,[43,46] and a chicken OVA sequence encoding a reporter epitope.[70,71] In the uniHELP construct, three N-terminal CD4$^+$ T cell antigens, separated by GPGPG-spacers, were added to the base construct: TTFCp30, PADRE, and HIV-nef$_{56-68}$.[37,38,72,73] In the neoHELP construct, three N-terminal CD4$^+$ T cell MC-38 neoantigens were added, also separated by GPGPG-spacers: Ddr2, Pcdh18, and Zmiz1.[48] In two additional neoHELP constructs, either the CD4$^+$ (neoHELP_CD4wt) or the CD8$^+$ (neoHELP_CD8wt) T cell neoantigens were reverted back to the wild-type sequences (see Table S1). All constructs also included a C-terminal HA-tag

(YPYDVPDYA) to enable quality control by flow cytometry and western blotting.

Co-transfection expression assay

B16-F10 (3,000 cells/well) or HEK293T (10,000 cells/well) cells were plated in 96-well flat-bottom plates (Greiner Bio-One, Kremsmünster, Austria) and transfected the next day with 25 ng of each of the indicated plasmids complexed with SAINT-DNA, according to the manufacturer's instructions (Synvolux Products, Leiden, the Netherlands). After 48 h, cells were trypsinized, washed, and fixed in 2% paraformaldehyde for 10 min at room temperature (RT). Cells were then permeabilized in FACS buffer (PBS with 0.5% bovine serum albumin [Sigma-Aldrich] and 0.01% sodium azide [LUMC] supplemented with 0.1% saponin [Sigma-Aldrich]) and subsequently stained with APC-conjugated anti-HA antibody (1:100, BioLegend, San Diego, CA, no. 901524). Fluorescence was measured using a Guava easyCyte flow cytometer and analyzed using the GuavaSoft 3.3 software (Luminex Corporation, Austin, TX).

Western blot

HEK293T cells (600,000 cells/well) were seeded in 6-well plates (Greiner Bio-One). The next day, cells were transfected with 500 ng of the indicated plasmids complexed with SAINT-DNA (Synvolux Products). Two days after transfection, cells were washed with ice-cold PBS, harvested, and centrifuged. Cell pellets were lysed in RIPA lysis buffer (1% IGEPAL, 0.15 M sodium chloride, 1% sodium deoxycholate, 1% SDS, 50 mM Tris-HCl [pH 8]) supplemented with protease inhibitor cocktail and benzonase nuclease (both from Sigma-Aldrich) for 30 min under constant agitation at 4°C. Subsequently, cell debris was removed by centrifugation at 16,000 $\times$ g for 20 min at 4°C. The protein concentrations of the cell lysates were measured using a BCA Protein assay (Thermo Scientific, Waltham, MA). Equal amounts of total proteins were loaded and separated by SDS-PAGE before transfer to a nitrocellulose membrane (GE Health, Chicago, IL). The membranes were blocked in PBS with 3% BSA and 0.5%, v/v, Tween 20 (PBS-T) overnight at 4°C, followed by 30 min at RT. They were then incubated for 1 h at RT with the primary antibody α-HA (1:500, BioLegend, no. 901513,) and subsequently with the secondary HRP-conjugated antibody HRP (1:2,000, BioLegend, no. 405306). Blots were developed with ECL substrate (GE Health) and analyzed using an ImageQuant LAS500 camera (GE Health).

In vitro antigen presentation assay

B16-F10 cells (30,000 cells/well) were plated in 96-well flat-bottom plates (Greiner Bio-One) and transfected with titrated amounts of plasmids complexed with SAINT-DNA (Synvolux Products). After 1 day, 50,000 B3Z reporter cells were added to each well. The next day, cells were treated with Z-buffer (0.18 mg/mL CPRG, 10 mM magnesium chloride, 0.125% IGEPAL, 0.1 M β-mercaptoethanol in PBS) to visualize β-galactosidase activity. Absorbance was measured at 594 nm using an Anthos Zenyth 3100 Multimode Fluorometer (Instrum, Marktheidenfeld, Germany).

Vaccinations and tissue collection

Using a 29G needle, naive mice were injected intradermally at the tail-base with 10 pmol of the linear DNA vaccines in 30 μL isotonic saline (0.9% NaCl) at 3-week intervals. For tetramer staining, blood was obtained via tail puncture and collected in heparinized tubes on days 13, 31, and 52 after the primary vaccination, and in some cases 13 days after tumor inoculation. For *ex vivo* functional T cell read-outs using spleen cells (ICS), mice were sacrificed 10 days after the final DNA vaccination (day 52).

ICS

D1 cells (100,000 cells/well) were seeded in 96-well-round bottom plates (Corning), and cultured with synthetic peptides (final concentration: 10 μg/mL) overnight (Table S2). The next day, spleens of vaccinated mice were collected in serum-free medium, and single cells were obtained by filtering the spleens over 70-μm cell strainers (BD, Franklin Lake, NJ), followed by erythrocyte lysis. Spleen cells (approximately 300,000 cells/well) were then cultured with the peptide-loaded D1 cells for 5 h, in the presence of 5 μg/mL brefeldin A (Sigma-Aldrich) during the final 4.5 h of incubation. Cells were stored overnight on ice. The next day, cells were first stained with fixable viability dye, followed by incubation with antibodies to CD3, CD4, and CD8 (Table S3). Subsequently, cells were fixed with fixation buffer (BioLegend) and stained with antibodies to CD40L, TNF, IL-2, and IFN-γ (Table S3) in Perm/Wash buffer (BioLegend). Cells were acquired on an LSRII (Becton Dickinson, CA), and the resulting data were analyzed using FlowJo software version 10.8.1 (Figures S2A and S2B).

Tetramer staining

Blood samples were exposed to erythrocyte lysis buffer (LUMC pharmacy, Leiden, the Netherlands) to remove red blood cells. Cells were washed with PBA (PBS [LUMC Pharmacy] supplemented with 0.1% bovine serum albumin [Sigma-Aldrich] and 0.02% sodium azide [LUMC Pharmacy]) and stained for 30 min at room temperature with tetramers: APC-conjugated H2-K^b/SIINFEKL or APC-conjugated H2-D^b/Rpl18 combined with PE-conjugated H2-D^b/Adpgk or PE-conjugated H2-D^b/Irgq (Table S3). After addition of antibodies to CD3, CD4, CD8, CD25, CX3CR1, KLRG1, PD-1, and LAG3, the cells were incubated for another 30 min. Cells were washed again with PBA before acquisition on an Aurora (Cytek, CA) spectral flow cytometer (Figures S2C, S2D, and S3) and resulting datasets were analyzed using FlowJo software version 10.8.1 (Tree Star, Ashland, OR).

Tumor challenge

Three weeks after the third vaccination (day 63), mice were injected subcutaneously with 300,000 MC-38 cells in 200 μL PBS in their right flank. Tumor growth was monitored using a caliper 2–3 times a week for 10 weeks after this challenge (until day 133). Mice were sacrificed when the tumor volume exceeded 1,000 mm^3. Tumor size (mm^3) was calculated by the following formula: tumor volume = length $\times$ width $\times$ width $\times$ 0.5. In selected experiments, $CD4^+$ or $CD8^+$ T cells were depleted by three intraperitoneal injections

(200 μL) of 100 μg of either α-CD4 (Bio X Cell, NH, clone GK1.5, BE0003-1) or α-CD8 (Bio X Cell, clone 2.43, BE0061) at days −6, −2, and +1 relative to the moment of tumor inoculation (days 57, 61, and 64).

Statistical analysis

Statistical analysis was performed using built-in methods from GraphPad Prism (version 8.0.1), as described in the figure legends. The resulting p values are indicated in the figures as follows: $p < 0.05$, $^{**}p < 0.01$, $^{***}p < 0.001$, $^{****}p < 0.0001$. Non-significant p values are not indicated.

DATA AND CODE AVAILABILITY

The data underlying Figures 1, 2, 3, 4, and 5 are available in the published article and its online supplemental information.

SUPPLEMENTAL INFORMATION

Supplemental information can be found online at https://doi.org/10.1016/j.omton.2024.200835.

ACKNOWLEDGMENTS

The authors are grateful to Yvonne de Vaal for technical assistance, and to Dr. Jannie Borst for critically reading the draft manuscript. This work was supported by grants from Health Holland, LSH-TKI project no. LSHM19076, and from the Netherlands Enterprise Agency (Rijksdienst voor Ondernemend Nederland, RVO), Innovation Credit IK17065.

AUTHOR CONTRIBUTIONS

J.F.d.G., T.P., F.S.S., and M.G.M.C. performed all animal and flow cytometry work. T.P. and K.O. performed initial flow cytometry protocol and panel validation, and I.Z., B.T., W.Z., and G.C.Z. produced linear DNA vaccines. J.F.d.G., T.A., R.A., J.v.B., and F.O. contributed to experimental design. All authors analyzed the data. J.v.B. and F.O. supervised the project. J.F.d.G., J.v.B., and F.O. wrote the manuscript.

DECLARATION OF INTERESTS

B.T. and G.C.Z. are inventors on patent application WO2020218924 – METHODS AND COMPOSITIONS FOR ISOTHERMAL DNA AMPLIFICATION, which describes the technology underlying the production of synthetic, linear DNA vaccines.

REFERENCES

1. Subudhi, S.K., Vence, L., Zhao, H., Blando, J., Yadav, S.S., Xiong, Q., Reuben, A., Aparicio, A., Corn, P.G., Chapin, B.F., et al. (2020). Neoantigen responses, immune correlates, and favorable outcomes after ipilimumab treatment of patients with prostate cancer. Sci. Transl. Med. *12*, eaaz3577.
2. McGranahan, N., Furness, A.J.S., Rosenthal, R., Ramskov, S., Lyngaa, R., Saini, S.K., Jamal-Hanjani, M., Wilson, G.A., Birkbak, N.J., Hiley, C.T., et al. (2016). Clonal neoantigens elicit T cell immunoreactivity and sensitivity to immune checkpoint blockade. Science *351*, 1463–1469.
3. Jardim, D.L., Goodman, A., de Melo Gagliato, D., and Kurzrock, R. (2021). The Challenges of Tumor Mutational Burden as an Immunotherapy Biomarker. Cancer Cell *39*, 154–173.
4. Chesney, J., Awasthi, S., Curti, B., Hutchins, L., Linette, G., Triozzi, P., Tan, M.C.B., Brown, R.E., Nemunaitis, J., Whitman, E., et al. (2018). Phase IIIb safety results from an expanded-access protocol of talimogene laherparepvec for patients with unresected, stage IIIB-IVM1c melanoma. Melanoma Res. *28*, 44–51.
5. Carreno, B.M., Magrini, V., Becker-Hapak, M., Kaabinejadian, S., Hundal, J., Petti, A.A., Ly, A., Lie, W.-R., Hildebrand, W.H., Mardis, E.R., and Linette, G.P. (2015). Cancer immunotherapy. A dendritic cell vaccine increases the breadth and diversity of melanoma neoantigen-specific T cells. Science *348*, 803–808.
6. Sahin, U., Derhovanessian, E., Miller, M., Kloke, B.-P., Simon, P., Löwer, M., Bukur, V., Tadmor, A.D., Luxemburger, U., Schrörs, B., et al. (2017). Personalized RNA mutanome vaccines mobilize poly-specific therapeutic immunity against cancer. Nature *547*, 222–226.
7. Ott, P.A., Hu, Z., Keskin, D.B., Shukla, S.A., Sun, J., Bozym, D.J., Zhang, W., Luoma, A., Giobbie-Hurder, A., Peter, L., et al. (2017). An immunogenic personal neoantigen vaccine for patients with melanoma. Nature *547*, 217–221.
8. Tondini, E., Arakelian, T., Oosterhuis, K., Camps, M., van Duikeren, S., Han, W., Arens, R., Zondag, G., van Bergen, J., and Ossendorp, F. (2019). A poly-neoantigen DNA vaccine synergizes with PD-1 blockade to induce T cell-mediated tumor control. OncoImmunology *8*, 1652539.
9. Shore, N.D., Morrow, M.P., McMullan, T., Kraynyak, K.A., Sylvester, A., Bhatt, K., Cheung, J., Boyer, J.D., Liu, L., Sacchetta, B., et al. (2020). $CD8^+$ T Cells Impact Rising PSA in Biochemically Relapsed Cancer Patients Using Immunotherapy Targeting Tumor-Associated Antigens. Mol. Ther. *28*, 1238–1250.
10. Keskin, D.B., Anandappa, A.J., Sun, J., Tirosh, I., Mathewson, N.D., Li, S., Oliveira, G., Giobbie-Hurder, A., Felt, K., Gjini, E., et al. (2019). Neoantigen vaccine generates intratumoral T cell responses in phase Ib glioblastoma trial. Nature *565*, 234–239.
11. Tanyi, J.L., Bobisse, S., Ophir, E., Tuyaerts, S., Roberti, A., Genolet, R., Baumgartner, P., Stevenson, B.J., Iseli, C., Dangaj, D., et al. (2018). Personalized cancer vaccine effectively mobilizes antitumor T cell immunity in ovarian cancer. Sci. Transl. Med. *10*, eaao5931.
12. Blass, E., and Ott, P.A. (2021). Advances in the development of personalized neoantigen-based therapeutic cancer vaccines. Nat. Rev. Clin. Oncol. *18*, 215–229.
13. Hillemanns, P., Denecke, A., Woelber, L., Böhmer, G., Jentschke, M., Schjetne, K.W., Bruins Slot, K.M.H., and Fredriksen, A.B. (2022). A Therapeutic Antigen-Presenting Cell-Targeting DNA Vaccine VB10.16 in HPV16-Positive High-Grade Cervical Intraepithelial Neoplasia: Results from a Phase I/IIa Trial. Clin. Cancer Res. *28*, 4885–4892.
14. Rojas, L.A., Sethna, Z., Soares, K.C., Olcese, C., Pang, N., Patterson, E., Lihm, J., Ceglia, N., Guasp, P., Chu, A., et al. (2023). Personalized RNA neoantigen vaccines stimulate T cells in pancreatic cancer. Nature *618*, 144–150.
15. Castle, J.C., Kreiter, S., Diekmann, J., Löwer, M., van de Roemer, N., de Graaf, J., Selmi, A., Diken, M., Boegel, S., Paret, C., et al. (2012). Exploiting the Mutanome for Tumor Vaccination. Cancer Res. *72*, 1081–1091.
16. Kreiter, S., Vormehr, M., van de Roemer, N., Diken, M., Löwer, M., Diekmann, J., Boegel, S., Schrörs, B., Vascotto, F., Castle, J.C., et al. (2015). Mutant MHC class II epitopes drive therapeutic immune responses to cancer. Nature *520*, 692–696.
17. Duperret, E.K., Perales-Puchalt, A., Stoltz, R., Mandloi, N., Mandloi, N., Barlow, J., Chaudhuri, A., Sardesai, N.Y., and Weiner, D.B. (2019). A Synthetic DNA, Multi-Neoantigen Vaccine Drives Predominately MHC Class I CD8+ T-cell Responses, Impacting Tumor Challenge. Cancer Immunol. Res. *7*, 174–182.
18. Weber, J.S., Carlino, M.S., Khattak, A., Meniawy, T., Ansstas, G., Taylor, M.H., Kim, K.B., McKean, M., Long, G.V., Sullivan, R.J., et al. (2024). Individualised neoantigen therapy mRNA-4157 (V940) plus pembrolizumab versus pembrolizumab monotherapy in resected melanoma (KEYNOTE-942): a randomised, phase 2b study. Lancet *403*, 632–644.
19. Nykode. (2021). Using an Inflammatory Chemokine Linked to the Antigen Generates a Rapid, Strong and Broad Immune Response. https://www.vaccibody.com/wp-content/uploads/2021/07/210722-Cell-mediated-therapies-for-infectious-diseases_final-004.pdf.
20. Bhojnagarwala, P.S., Perales-Puchalt, A., Cooch, N., Sardesai, N.Y., and Weiner, D.B. (2021). A synDNA vaccine delivering neoAg collections controls heterogenous, multifocal murine lung and ovarian tumors via robust T cell generation. Mol. Ther. Oncolytics *21*, 278–287.

21. Eickhoff, S., Brewitz, A., Gerner, M.Y., Klauschen, F., Komander, K., Hemmi, H., Garbi, N., Kaisho, T., Germain, R.N., and Kastenmüller, W. (2015). Robust Anti-viral Immunity Requires multiple distinct T cell-dendritic cell interactions. Cell *162*, 1322–1337.
22. Hor, J.L., Whitney, P.G., Zaid, A., Brooks, A.G., Heath, W.R., and Mueller, S.N. (2015). Spatiotemporally Distinct Interactions with Dendritic Cell Subsets Facilitates $CD4^+$ and $CD8^+$ T Cell Activation to Localized Viral Infection. Immunity *43*, 554–565.
23. Schoenberger, S.P., Toes, R.E., van der Voort, E.I., Offringa, R., and Melief, C.J. (1998). T-cell help for cytotoxic T lymphocytes is mediated by CD40–CD40L interactions. Nature *393*, 480–483.
24. Bennett, S.R., Carbone, F.R., Karamalis, F., Flavell, R.A., Miller, J.F., and Heath, W.R. (1998). Help for cytotoxic-T-cell responses is mediated by CD40 signalling. Nature *393*, 478–480.
25. Ridge, J.P., Di Rosa, F., and Matzinger, P. (1998). A conditioned dendritic cell can be a temporal bridge between a CD4+ T-helper and a T-killer cell. Nature *393*, 474–478.
26. Williams, M.A., Tyznik, A.J., and Bevan, M.J. (2006). Interleukin-2 signals during priming are required for secondary expansion of CD8+ memory T cells. Nature *441*, 890–893.
27. Ahrends, T., Spanjaard, A., Pilzecker, B., Bąbała, N., Bovens, A., Xiao, Y., Jacobs, H., and Borst, J. (2017). CD4+ T Cell Help Confers a Cytotoxic T Cell Effector Program Including Coinhibitory Receptor Downregulation and Increased Tissue Invasiveness. Immunity *47*, 848–861.e5.
28. Ahrends, T., Busselaar, J., Severson, T.M., Bąbała, N., de Vries, E., Bovens, A., Wessels, L., van Leeuwen, F., and Borst, J. (2019). CD4+ T cell help creates memory CD8+ T cells with innate and help-independent recall capacities. Nat. Commun. *10*, 5531.
29. Shedlock, D.J., and Shen, H. (2003). Requirement for CD4 T Cell Help in Generating Functional CD8 T Cell Memory. Science *300*, 337–339.
30. Sun, J.C., and Bevan, M.J. (2003). Defective CD8 T Cell Memory Following Acute Infection Without CD4 T Cell Help. Science *300*, 339–342.
31. Janssen, E.M., Lemmens, E.E., Wolfe, T., Christen, U., von Herrath, M.G., and Schoenberger, S.P. (2003). CD4+ T cells are required for secondary expansion and memory in CD8+ T lymphocytes. Nature *421*, 852–856.
32. Ossendorp, F., Mengedé, E., Camps, M., Filius, R., and Melief, C.J. (1998). Specific T Helper Cell Requirement for Optimal Induction of Cytotoxic T Lymphocytes against Major Histocompatibility Complex Class II Negative Tumors. J. Exp. Med. *187*, 693–702.
33. Alspach, E., Lussier, D.M., Miceli, A.P., Kizhvatov, I., DuPage, M., Luoma, A.M., Meng, W., Lichti, C.F., Esaulova, E., Vomund, A.N., et al. (2019). MHC-II neoantigens shape tumour immunity and response to immunotherapy. Nature *574*, 696–701.
34. Tran, E., Turcotte, S., Gros, A., Robbins, P.F., Lu, Y.-C., Dudley, M.E., Wunderlich, J.R., Somerville, R.P., Hogan, K., Hinrichs, C.S., et al. (2014). Cancer Immunotherapy Based on Mutation-Specific CD4+ T Cells in a Patient with Epithelial Cancer. Science *344*, 641–645.
35. Veatch, J.R., Lee, S.M., Fitzgibbon, M., Chow, I.-T., Jesernig, B., Schmitt, T., Kong, Y.Y., Kargl, J., Houghton, A.M., Thompson, J.A., et al. (2018). Tumor-infiltrating BRAFV600E-specific CD4+ T cells correlated with complete clinical response in melanoma. J. Clin. Invest. *128*, 1563–1568.
36. Andreatta, M., Karosiene, E., Rasmussen, M., Stryhn, A., Buus, S., and Nielsen, M. (2015). Accurate pan-specific prediction of peptide-MHC class II binding affinity with improved binding core identification. Immunogenetics *67*, 641–650.
37. Alexander, J., Sidney, J., Southwood, S., Ruppert, J., Oseroff, C., Maewal, A., Snoke, K., Serra, H.M., Kubo, R.T., Sette, A., et al. (1994). Development of high potency universal DR-restricted helper epitopes by modification of high affinity DR-blocking peptides. Immunity *1*, 751–761.
38. Cohen, W.M., Pouvelle-Moratille, S., Wang, X.-F., Farci, S., Munier, G., Charron, D., Ménez, A., Busson, M., and Maillère, B. (2006). Scanning the HIV Genome for CD4 T Cell Epitopes Restricted to HLA-DP4, the Most Prevalent HLA Class II Molecule. J. Immunol. *176*, 5401–5408.
39. Oosterhuis, K., Aleyd, E., Vrijland, K., Schumacher, T.N., and Haanen, J.B. (2012). Rational Design of DNA Vaccines for the Induction of Human Papillomavirus Type 16 E6- and E7-Specific Cytotoxic T-Cell Responses. Hum. Gene Ther. *23*, 1301–1312.
40. Xu, Z., Chokkalingam, N., Tello-Ruiz, E., Walker, S., Kulp, D.W., and Weiner, D.B. (2020). Incorporation of a Novel CD4+ Helper Epitope Identified from Aquifex aeolicus Enhances Humoral Responses Induced by DNA and Protein Vaccinations. iScience *23*, 101399.
41. Lu, Y.-J., Barreira-Silva, P., Boyce, S., Powers, J., Cavallo, K., and Behar, S.M. (2021). CD4 T cell help prevents CD8 T cell exhaustion and promotes control of Mycobacterium tuberculosis infection. Cell Rep. *36*, 109696.
42. Koutsoumpli, G., Ip, P.P., Schepel, I., Hoogeboom, B.N., Boerma, A., and Daemen, T. (2019). Alphavirus-based hepatitis C virus therapeutic vaccines: can universal helper epitopes enhance HCV-specific cytotoxic T lymphocyte responses? Ther. Adv. Vaccines Immunother. *7*, 2515135519874677.
43. Yadav, M., Jhunjhunwala, S., Phung, Q.T., Lupardus, P., Tanguay, J., Bumbaca, S., Franci, C., Cheung, T.K., Fritsche, J., Weinschenk, T., et al. (2014). Predicting immunogenic tumour mutations by combining mass spectrometry and exome sequencing. Nature *515*, 572–576.
44. Aurisicchio, L., Salvatori, E., Lione, L., Bandini, S., Pallocca, M., Maggio, R., Fanciulli, M., De Nicola, F., Goeman, F., Ciliberto, G., et al. (2019). Poly-specific neoantigen-targeted cancer vaccines delay patient derived tumor growth. J. Exp. Clin. Cancer Res. *38*, 78.
45. Capietto, A.-H., Jhunjhunwala, S., Pollock, S.B., Lupardus, P., Wong, J., Hänsch, L., Cevallos, J., Chestnut, Y., Fernandez, A., Lounsbury, N., et al. (2020). Mutation position is an important determinant for predicting cancer neoantigens. J. Exp. Med. *217*, e20190179.
46. Hos, B.J., Camps, M.G., Bulk, J.v.d., Tondini, E., Ende, T.C.v.d., Ruano, D., Franken, K., Janssen, G.M., Ru, A., Filippov, D.V., et al. (2020). Identification of a neo-epitope dominating endogenous CD8 T cell responses to MC-38 colorectal cancer. OncoImmunology *9*, 1673125.
47. Leoni, G., D'Alise, A.M., Tucci, F.G., Micarelli, E., Garzia, I., De Lucia, M., Langone, F., Nocchi, L., Cotugno, G., Bartolomeo, R., et al. (2021). VENUS, a Novel Selection Approach to Improve the Accuracy of Neoantigens' Prediction. Vaccines *9*, 880. https://doi.org/10.3390/vaccines9080880.
48. Hos, B.J., Tondini, E., Camps, M.G.M., Rademaker, W., van den Bulk, J., Ruano, D., Janssen, G.M.C., de Ru, A.H., van den Elsen, P.J., de Miranda, N.F.C.C., et al. (2022). Cancer-specific T helper shared and neo-epitopes uncovered by expression of the MHC class II master regulator CIITA. Cell Rep. *41*, 111680.
49. Pollock, S.B., Rose, C.M., Darwish, M., Bouziat, R., Delamarre, L., Blanchette, C., and Lill, J.R. (2021). Sensitive and Quantitative Detection of MHC-I Displayed Neoepitopes Using a Semiautomated Workflow and TOMAHAQ Mass Spectrometry. Mol. Cell. Proteomics *20*, 100108.
50. Panagioti, E., Redeker, A., van Duikeren, S., Franken, K.L., Drijfhout, J.W., van der Burg, S.H., and Arens, R. (2016). The Breadth of Synthetic Long Peptide Vaccine-Induced CD8+ T Cell Responses Determines the Efficacy against Mouse Cytomegalovirus Infection. PLoS Pathog. *12*, e1005895.
51. Olson, J.A., McDonald-Hyman, C., Jameson, S.C., and Hamilton, S.E. (2013). Effector-like $CD8^+$ T Cells in the Memory Population Mediate Potent Protective Immunity. Immunity *38*, 1250–1260.
52. Beyrend, G., van der Gracht, E., Yilmaz, A., van Duikeren, S., Camps, M., Höllt, T., Vilanova, A., van Unen, V., Koning, F., de Miranda, N.F.C.C., et al. (2019). PD-L1 blockade engages tumor-infiltrating lymphocytes to co-express targetable activating and inhibitory receptors. J. Immunother. Cancer *7*, 217.
53. Borst, J., Ahrends, T., Bąbała, N., Melief, C.J.M., and Kastenmüller, W. (2018). CD4+ T cell help in cancer immunology and immunotherapy. Nat. Rev. Immunol. *18*, 635–647.
54. Wong, S.B.J., Bos, R., and Sherman, L.A. (2008). Tumor-Specific $CD4^+$ T Cells Render the Tumor Environment Permissive for Infiltration by Low-Avidity CD8 T Cells. J. Immunol. *180*, 3122–3131.
55. Bos, R., and Sherman, L.A. (2010). CD4+ T-Cell Help in the Tumor Milieu Is Required for Recruitment and Cytolytic Function of CD8+ T Lymphocytes. Cancer Res. *70*, 8368–8377.
56. Hofer, T., Rossi, M., Carboni, S., Di Berardino Besson, W., von Laer, D., Wollmann, G., Derouazi, M., and Santiago-Raber, M.-L. (2021). Heterologous Prime-Boost

Vaccination with a Peptide-Based Vaccine and Viral Vector Reshapes Dendritic Cell, CD4+ and CD8+ T Cell Phenotypes to Improve the Antitumor Therapeutic Effect. Cancers *13*, 6107.

57. Mumberg, D., Monach, P.A., Wanderling, S., Philip, M., Toledano, A.Y., Schreiber, R.D., and Schreiber, H. (1999). CD4(+) T cells eliminate MHC class II-negative cancer cells in vivo by indirect effects of IFN-gamma. Proc. Natl. Acad. Sci. USA *96*, 8633–8638.

58. Brightman, S.E., Becker, A., Thota, R.R., Naradikian, M.S., Chihab, L., Zavala, K.S., Griswold, R.Q., Dolina, J.S., Cohen, E.E.W., Miller, A.M., et al. (2023). Neoantigen-specific stem cell memory-like CD4+ T cells mediate CD8+ T cell-dependent immunotherapy of MHC class II-negative solid tumors. Nat. Immunol. *24*, 1345–1357.

59. Dolina, J.S., Lee, J., Brightman, S.E., McArdle, S., Hall, S.M., Thota, R.R., Lanka, M., Premlal, A.L.R., Greenbaum, J.A., Cohen, E.E.W., et al. (2023). Linked CD4 +/CD8 + T cell neoantigen vaccination overcomes immune checkpoint blockade resistance and enables tumor regression. J Clin. Invest. *133*, e164258.

60. Aarntzen, E.H.J.G., De Vries, I.J.M., Lesterhuis, W.J., Schuurhuis, D., Jacobs, J.F.M., Bol, K., Schreibelt, G., Mus, R., De Wilt, J.H.W., Haanen, J.B.A.G., et al. (2013). Targeting CD4+ T-Helper Cells Improves the Induction of Antitumor Responses in Dendritic Cell–Based Vaccination. Cancer Res. *73*, 19–29.

61. Sabarth, N., Chamberlain, L., Brett, S., Tite, J., and Craigen, J. (2010). Induction of Homologous Rather than Heterologous Antigen-Specific CD4 T Cell Responses Is Critical for Functional CD8 T Cell Responses in Mice Transgenic for a Foreign Antigen. J. Immunol. *185*, 4590–4601.

62. Attermann, A.S., Barra, C., Reynisson, B., Schultz, H.S., Leurs, U., Lamberth, K., and Nielsen, M. (2021). Improved prediction of HLA antigen presentation hotspots: Applications for immunogenicity risk assessment of therapeutic proteins. Immunology *162*, 208–219.

63. Forlani, G., Michaux, J., Pak, H., Huber, F., Marie Joseph, E.L., Ramia, E., Stevenson, B.J., Linnebacher, M., Accolla, R.S., and Bassani-Sternberg, M. (2021). CIITA-Transduced Glioblastoma Cells Uncover a Rich Repertoire of Clinically Relevant Tumor-Associated HLA-II Antigens. Mol. Cell. Proteomics *20*, 100032.

64. Racle, J., Michaux, J., Rockinger, G.A., Arnaud, M., Bobisse, S., Chong, C., Guillaume, P., Coukos, G., Harari, A., Jandus, C., et al. (2019). Robust prediction of HLA class II epitopes by deep motif deconvolution of immunopeptidomes. Nat. Biotechnol. *37*, 1283–1286.

65. Chen, B., Khodadoust, M.S., Olsson, N., Wagar, L.E., Fast, E., Liu, C.L., Muftuoglu, Y., Sworder, B.J., Diehn, M., Levy, R., et al. (2019). Predicting HLA class II antigen presentation through integrated deep learning. Nat. Biotechnol. *37*, 1332–1343.

66. Abelin, J.G., Harjanto, D., Malloy, M., Suri, P., Colson, T., Goulding, S.P., Creech, A.L., Serrano, L.R., Nasir, G., Nasrullah, Y., et al. (2019). Defining HLA-II Ligand Processing and Binding Rules with Mass Spectrometry Enhances Cancer Epitope Prediction. Immunity *51*, 766–779.e17.

67. Rappazzo, C.G., Huisman, B.D., and Birnbaum, M.E. (2020). Repertoire-scale determination of class II MHC peptide binding via yeast display improves antigen prediction. Nat. Commun. *11*, 4414.

68. Zondag, G., Teunisse, B., and Paolini, N. (2020). Methods and Compositions for Isothermal DNA Amplification. WO/2020/218924.

69. Allen, A., Wang, C., Caproni, L.J., Sugiyarto, G., Harden, E., Douglas, L.R., Duriez, P.J., Karbowniczek, K., Extance, J., Rothwell, P.J., et al. (2018). Linear doggybone DNA vaccine induces similar immunological responses to conventional plasmid DNA independently of immune recognition by TLR9 in a pre-clinical model. Cancer Immunol. Immunother. *67*, 627–638.

70. Falo, L.D., Kovacsovics-Bankowski, M., Thompson, K., and Rock, K.L. (1995). Targeting antigen into the phagocytic pathway in vivo induces protective tumour immunity. Nat. Med. *1*, 649–653.

71. Moore, M.W., Carbone, F.R., and Bevan, M.J. (1988). Introduction of soluble protein into the class I pathway of antigen processing and presentation. Cell *54*, 777–785.

72. Panina-Bordignon, P., Tan, A., Termijtelen, A., Demotz, S., Corradin, G., and Lanzavecchia, A. (1989). Universally immunogenic T cell epitopes: promiscuous binding to human MHC class II and promiscuous recognition by T cells. Eur. J. Immunol. *19*, 2237–2242.

73. Swartz, A.M., Congdon, K.L., Nair, S.K., Li, Q.-J., Herndon, J.E., Suryadevara, C.M., Riccione, K.A., Archer, G.E., Norberg, P.K., Sanchez-Perez, L.A., and Sampson, J.H. (2021). A conjoined universal helper epitope can unveil antitumor effects of a neoantigen vaccine targeting an MHC class I-restricted neoepitope. NPJ Vaccines *6*, 12.

OMTON, Volume *32*

Supplemental information

Neoantigen-specific T cell help outperforms non-specific help in multi-antigen DNA vaccination against cancer

Joanna Fréderique de Graaf, Tamara Pesic, Felicia S. Spitzer, Koen Oosterhuis, Marcel G.M. Camps, Iris Zoutendijk, Bram Teunisse, Wahwah Zhu, Tsolere Arakelian, Gerben C. Zondag, Ramon Arens, Jeroen van Bergen, and Ferry Ossendorp

Supplemental information

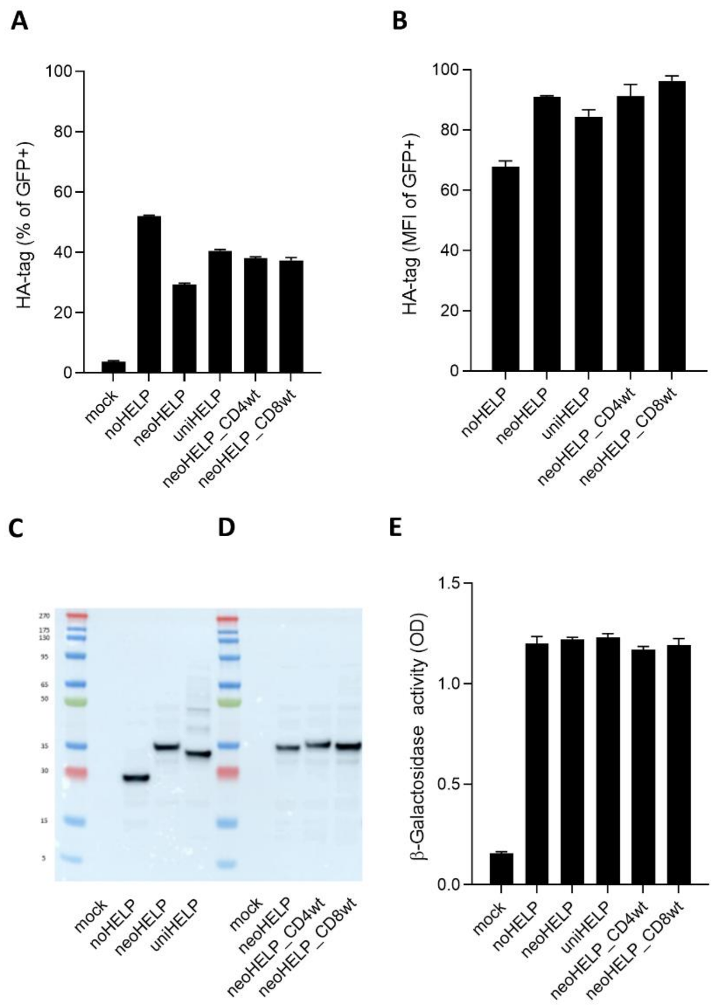

Figure S1. Verification of vaccine constructs. (A-B) HEK293T cells were transfected with a GFP-encoding plasmid in combination with the indicated vaccine-encoding plasmids. Transfected cells were identified by GFP-expression, and a C-terminal HA-tag allowed detection of multi-antigen vaccine proteins. (A) The fraction (%) of HA-positive cells among transfected (GFP^+) cells, and (B) their mean fluorescence intensity (MFI) were determined by flow cytometry. The MFI of mock-transfected cells is not shown as these cells – not having been transfected with a plasmid encoding an HA-tagged protein - do not have detectable expression of the HA-tag above background. Bars and whiskers represent means and standard errors (SEM) of triplicates, respectively. (C-D) Western blot detection of HA-tagged (C) noHELP, neoHELP, uniHELP or (D) neoHELP, neoHELP_CD4wt or neoHELP_CD8wt in HEK293T cells transfected with the indicated vaccine-encoding plasmids. (E) Recognition of OVA antigen on transfected B16-F10 cells by H-$2K^b$/OVA-specific $CD8^+$ T-cell hybridoma B3Z. β-galactosidase expression in B3Z cells is controlled by NFAT, allowing detection of TCR-mediated activation by color conversion of CPRG substrate, which results in increased optical density (OD) at 594nm. Bars and whiskers represent means and standard errors (SEM) of triplicates, respectively. All experiments were performed twice, and one representative experiment is shown.

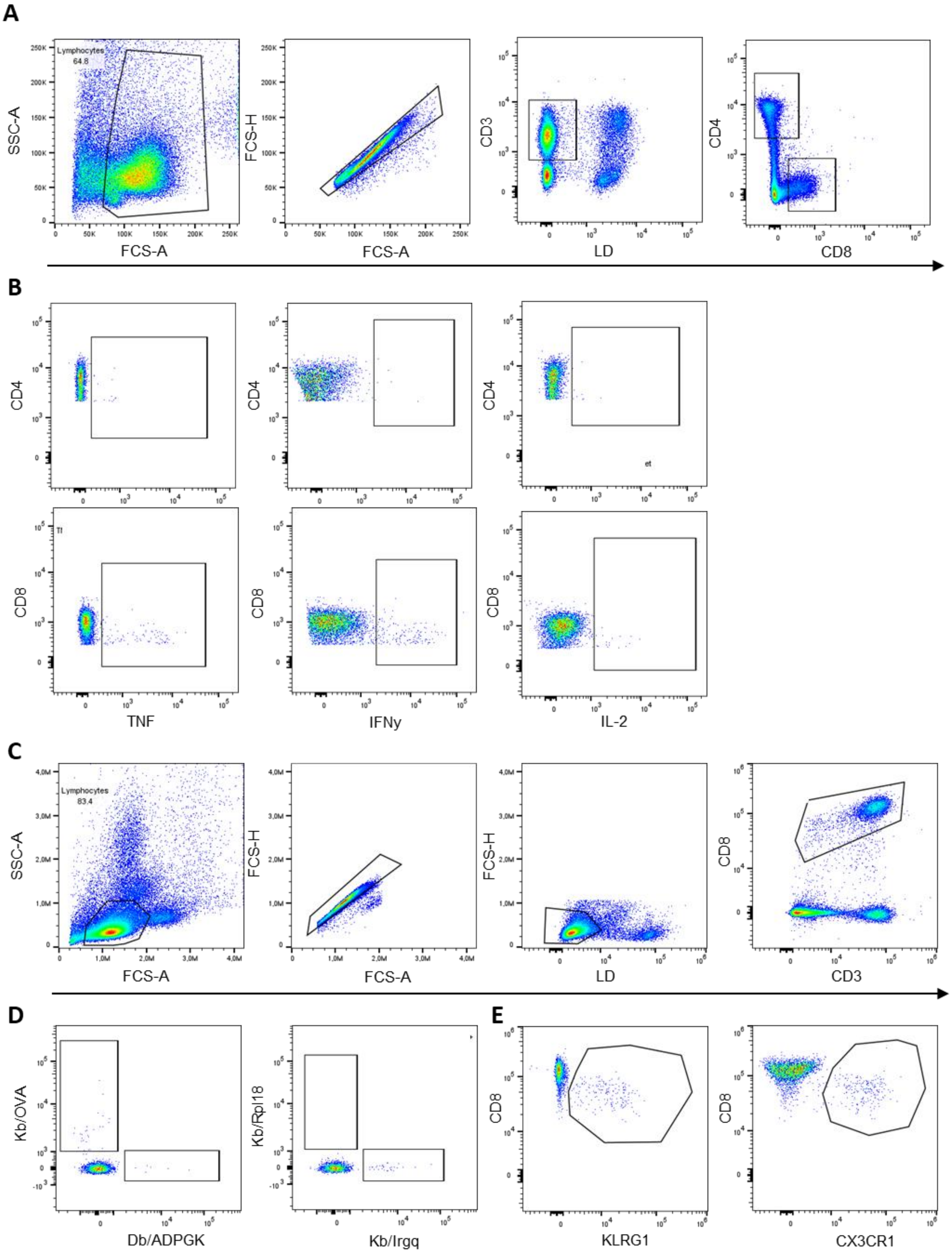

Figure S2. Gating strategy for flow cytometry analysis. (A-B) Gating strategy for spleen-derived T-lymphocytes for ICS. (A) Live CD4⁺ and CD8⁺ T-cells were gated after which (B) TNF-, IFN-γ- and IL-2-positive cells were selected. (C-E) Gating strategy for antigen-specific peripheral blood- and spleen-derived T-lymphocytes for surface marker analysis. (C) Gating of live CD8⁺ T-cells was followed by (D) selection of tetramer-positive cells. (E) Subsequently, cells positive for specific surface markers were identified.

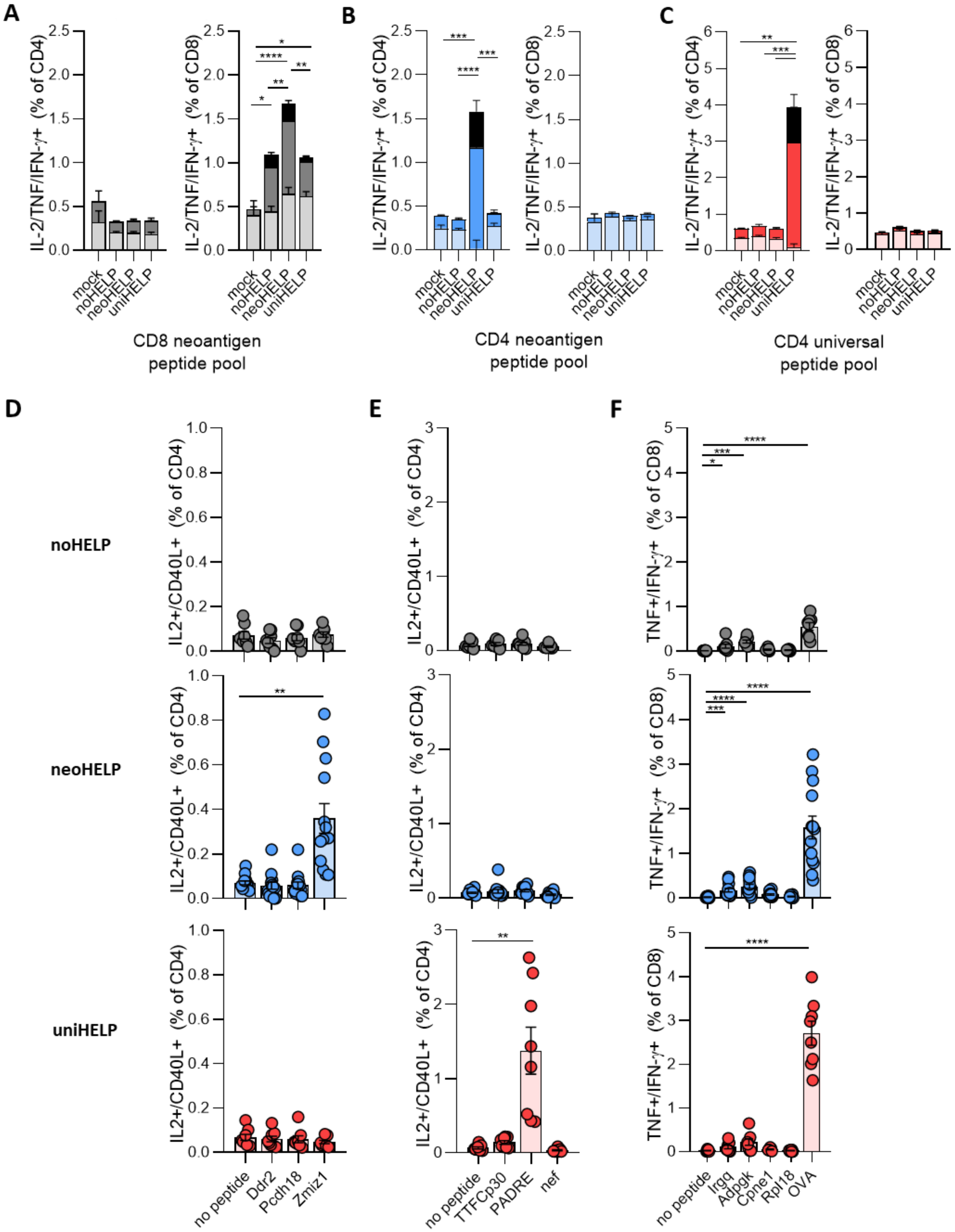

Figure S3. Antigen-specific CD4⁺ and CD8⁺ T-cell responses in the spleen of mice. Mice were vaccinated with the indicated vaccines three times, at three-week intervals. Ten days after the final vaccination, spleen cells were cultured with dendritic cells loaded with indicated (A-C) peptide pools or (D-F) individual peptides for 5 hours, and analyzed by intracellular cytokine staining (ICS). IL-2-, TNF-

and/or IFN-γ-positive $CD4^+$ or $CD8^+$ T-cells upon stimulation with the indicated peptide pools: (A) CD8 neoantigen peptide pool (Irgq, Adpgk, Cpne1, Rpl18), (B) CD4 neoantigen peptide pool (Ddr2, Pcdh18, Zmiz1), (C) CD4 universal peptide pool (TTFCp30, PADRE, nef). Data are derived from a single experiment with 5 (mock, noHELP, uniHELP) or 10 (neoHELP) mice per group. Data in A-C were analyzed by a two-way ANOVA test followed by Tukey's multiple comparisons test. *p<0.05, **p<0.01, ***p<0.001, ****p<0.0001. (D) Fraction of $CD4^+$ T-cells expressing IL-2 and CD40L in response to tumor-specific neoantigens Ddr1, Pdch1 and Zmiz1. (E) Fraction of $CD4^+$ T-cells expressing IL-2 and CD40L in response to tumor-unrelated 'universal' antigens TTFC30, PADRE and HIV-nef58-68 (nef). (F) Fraction of $CD8^+$ T-cells expressing TNF and IFN-γ in response to tumor-specific neoantigens Irgq, Adpgk, Cpne1,Rpl18 and to an ovalbumin-derived reporter antigen (OVA). Data are derived from two independent experiments with 8 (mock, noHELP, uniHELP) or 13 (neoHELP) mice per group in total. Dots represent individual values, bars and whiskers represent means and standard errors (SEM), respectively. Data in D-F were analyzed by a Kruskal-Wallis test followed by Dunnet's multiple comparisons test. *p<0.05, **p<0.01, ***p<0.001, ****p<0.0001.

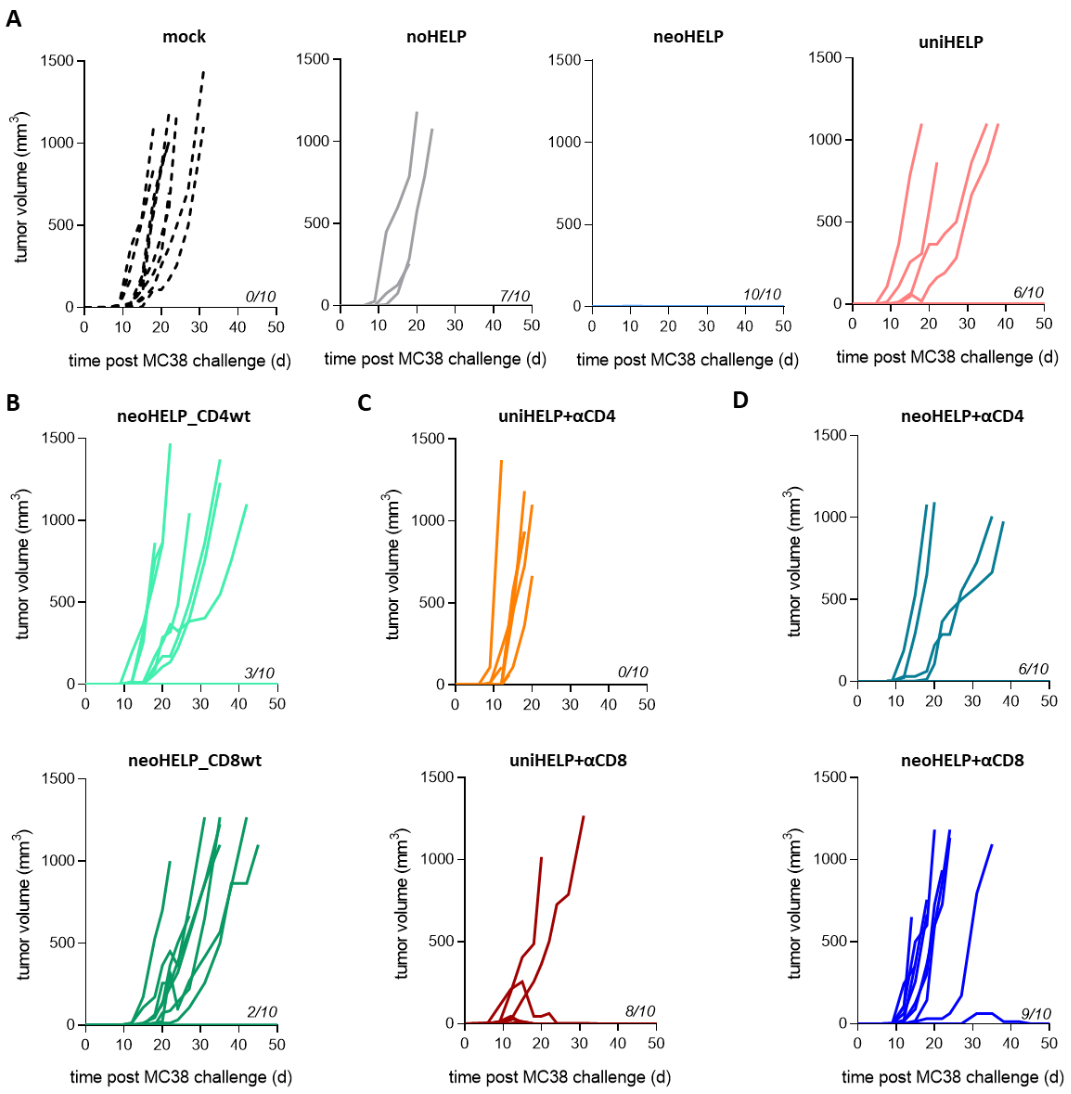

Figure S4. Tumor growth upon depletion of CD4 and CD8 responses. Mice (10 mice per group) were vaccinated three times, at 3-week intervals, with the indicated vaccines. Selected groups were depleted of $CD4^+$ or $CD8^+$ T-cells by three injections of 100 µg of either α-CD4 (clone GK1.5) or α-CD8 (clone 2.43) on days 57, 61 and 64 post primary vaccination. On day 63 post primary vaccination, mice were injected subcutaneously with 300.000 MC-38 colon carcinoma cells. Lengths and widths of the tumors were measured multiple times a week with a digital caliper to calculate tumor volume. Mice were removed from the experiment if the tumor exceeded 1000 mm^3 or if an ulcer occurred. Mice were vaccinated with (A) saline solution (mock), uniHELP or neoHELP (B) neoHELP vaccines carrying the wild-type counterparts of either the CD4 (neoHELP_CD4wt) or CD8 (neoHELP CD4wt) T-cell antigens. Additional groups of mice vaccinated with (C) uniHELP and (D) neoHELP underwent $CD4^+$ or $CD8^+$ T-cell depletion around the time of tumor challenge. Tumor volumes of individual mice are plotted, and the numbers of tumor-free mice at day 50 post challenge are indicated at the bottom right of each graph.

Table S1. $CD8^+$ and $CD4^+$ T-cell antigens used DNA vaccines

Antigen	Gene	Amino Acid (AA) sequence*	wt>mt (AA)	MHC-restriction
Irgq[43,46]	*Irgq*	QNAAKARDET**AALLNSAVL**GAAPLFVPPADCSSSD	G>V	H2-D^b
Adpgk[43–46,49]	*Adpgk*	DIPTGIPVHLEL**ASMTNMELM**SSIVHQQVFPTVAS	R>M	H2-D^b
Cpne1[43,46,49]	*Cpne1*	FTVGVDFTGSNGDP**SSPYSLHYL**SPTGVNEYLTAL	D>Y	H2-D^b
Rpl18[46,49]	*Rpl18*	RARSRILKAGG**KILTFDRL**ALESPKGRGTVLLSGP	Q>R	H2-D^b
OVA[67,68]	*SERPINB14*	LLPDEVSGLEQLE**SIINFEKL**TEWTSSNVMEERKI	n.a.	H2-K^b
Ddr2[48]	*Ddr2*	FKEVQCYFR**SEASEWEPHAVYFPLVLDDVNPS**ARF	T>H	I-A^b
Pcdh18[48]	*Pdhc18*	RFQRSRYEFVISENN**SPWAYITTVTATDPDL**GENG	G>W	I-A^b
Zmiz1[48]	*Zmiz1*	GIPPHT**RPPADFTQPAASAAAAA**VAAAAATATATA	A>S	I-A^b
TTFCp30[69,70]	*tetX*	**FNNFTVSFWLRVPKVSASHLE**	n.a.	I-A^b
PADRE[37]	*n.a.*	**AKFVAAWTLKAAA**	n.a.	I-A^b
HIV nef[38]	*nef*	**AWLEAQEEEEVGF**	n.a.	-

* T-cell epitopes in **boldface**, mutated residues underlined.

Table S2. Synthetic peptides used for intracellular cytokine staining (ICS) experiments

Antigen	Amino Acid (AA) sequence	Source
Irgq	RDETAALLNSAVLGAAPLFV	LUMC-Immunology synthetic peptide facility
Adpgk	ELASMTNMELMSSIV	LUMC-Immunology synthetic peptide facility
Cpne1	GSNGDPSSPYSLHYLSPTGVNE	LUMC-Immunology synthetic peptide facility
Rpl18	KAGGKILTFDRLALESPK	LUMC-Immunology synthetic peptide facility
OVA[67,68]	DEVSGLEQLESIINFEKLAAAAAK	LUMC-Immunology synthetic peptide facility
Ddr2	SEASEWEPHAVYFPLVLDDVNPS	LUMC-Immunology synthetic peptide facility
Pcdh18[48]	SPWAYITTVTATDPDL	LUMC-Immunology synthetic peptide facility
Zmiz1[48]	RPPADFTQPAASAAAAA	LUMC-Immunology synthetic peptide facility
TTFCp30	FNNFTVSFWLRVPKVSASHLE	LUMC-Immunology synthetic peptide facility
PADRE	AKFVAAWTLKAAA	LUMC-Immunology synthetic peptide facility
HIV nef	AWLEAQEEEEVGF	LUMC-Immunology synthetic peptide facility

Table S3. Flow cytometry antibody list

Antibodies	Fluorochrome	Source	Identifier
Fixable Viability Dye	eF450	Thermo Fisher	65-0863-14
Fixable Viability Dye	APC-eF780	Thermo Fisher	65-0865-14
CD3 (clone: 145-2C11)	BV510	BioLegend	100353
CD3 (clone: 145-2C11)	APC	Thermo Fisher	17-0031-83
CD4 (clone: RM4-5)	BV711	BioLegend	100550
CD4 (clone: GK1.5)	AF700	Thermo Fisher	56-0041-82
CD4 (clone: RM4-5)	PE-Cy7	Invitrogen	25-0042-82
CD8α (clone: 53-6.7)	APC-eF780	BioLegend	100744
CD8α (clone: 53-6.7)	APC-R700	BD Sciences	564983
CD8α (clone: 53-6.7)	BUV805	BD Horizon	612898
CD25 (clone: PC61)	BV711	BioLegend	102049
PD-1 (clone: 29F.1A12)	BV605	BioLegend	125225
LAG3 (clone: C9B7W)	PE-Cy7	Thermo Fisher	12-9942-81
CX3CR1 (clone: SA011F11)	BV785	Thermo Fisher	35-5893-82
KLRG1 (clone: 2F1)	FITC	BioLegend	138409
IL-2 (clone: JES6-5H4)	APC	BD Biosciences	554429
CD40L (clone: MR1)	PE	Thermo Fisher	12-1541-82
TNF (clone: MP6-XT22)	FITC	BioLegend	506304
IFNγ (clone: XMG1.2)	PE-Cy7	BD Biosciences	557649
H2-K^b/SIINFEKL	APC	LUMC-Immunology	tetramer facility
H2-K^b/Rpl18	APC	LUMC-Immunology	tetramer facility
H2-D^b/Adpgk	PE	LUMC-Immunology	tetramer facility
H2-D^b/Irgq	PE	LUMC-Immunology	tetramer facility

Table S4. Raw data of figures
